# Coordinated Regulation of Niche and Stem Cell Precursors by Hormonal Signaling

**DOI:** 10.1371/journal.pbio.1001202

**Published:** 2011-11-22

**Authors:** Dana Gancz, Tamar Lengil, Lilach Gilboa

**Affiliations:** Department of Biological Regulation, Weizmann Institute of Science, Rehovot, Israel; University of Edinburgh, United Kingdom

## Abstract

Stem cells and their niches constitute units that act cooperatively to achieve adult body homeostasis. How such units form and whether stem cell and niche precursors might be coordinated already during organogenesis are unknown. In fruit flies, primordial germ cells (PGCs), the precursors of germ line stem cells (GSCs), and somatic niche precursors develop within the larval ovary. Together they form the 16–20 GSC units of the adult ovary. We show that ecdysone receptors are required to coordinate the development of niche and GSC precursors. At early third instar, ecdysone receptors repress precocious differentiation of both niches and PGCs. Early repression is required for correct morphogenesis of the ovary and for protecting future GSCs from differentiation. At mid-third instar, ecdysone signaling is required for niche formation. Finally, and concurrent with the initiation of wandering behavior, ecdysone signaling initiates PGC differentiation by allowing the expression of the differentiation gene *bag of marbles* in PGCs that are not protected by the newly formed niches. All the ovarian functions of ecdysone receptors are mediated through early repression, and late activation, of the ecdysone target gene *broad*. These results show that, similar to mammals, a brain-gland-gonad axis controls the initiation of oogenesis in insects. They further exemplify how a physiological cue coordinates the formation of a stem cell unit within an organ: it is required for niche establishment and to ensure that precursor cells to adult stem cells remain undifferentiated until the niches can accommodate them. Similar principles might govern the formation of additional stem cell units during organogenesis.

## Abstract 2

In the developing *Drosophila* ovary, the ecdysone signaling pathway controls the differentiation of both niche and germ line stem cell precursors.

## Author Summary

Tissue-specific stem cells reside in specialized microenvironments (niches). How the generation of niche cells and resident stem cells is coordinated, and how their correct numerical ratios are regulated, remains poorly understood. Here, we examine the potential mechanisms of this process by analyzing the formation of the fly ovary. Specifically, we uncover a role for ecdysone, which is a steroid hormone renowned for its role in insect molting but that also controls many aspects of larval development in flies and mammals. We find that ecdysone signaling in fly larvae coordinates the development of niche cells relative to their resident germ line stem cells (GSCs). Ecdysone receptors initially serve as repressors of differentiation, allowing precursor cells of both niches and GSCs time to proliferate and attain correct cell numbers. Later, ecdysone receptors allow differentiation of niches while simultaneously maintaining GSC precursors in an undifferentiated state, until the newly formed niches can accommodate them. Finally, ecdysone induces the differentiation of GSC precursors that are not incorporated in niches. Our work exemplifies one possible solution to coordinating stem cell and niche development: using a common signal to affect both cell types. A further understanding of these and other mechanisms will offer novel insights into regeneration and could help guide cell-based therapies for various diseases.

## Introduction

Stem cells and their niches constitute functional units that underlie adult organ homeostasis and regeneration following injury or disease. Despite their great medical importance, little is known about how stem cell units, which originate from precursor cells, form during development. Understanding the relations between stem cell precursors and niche precursors and uncovering the molecular pathways that govern the behavior of these populations are likely to enhance our potential to use stem cells in cell-based therapies. Here we use the developing ovary of the fruit fly *Drosophila melanogaster* as a model to investigate how the formation of niches is coordinated with the development of their resident stem cells.

**Figure 1:**
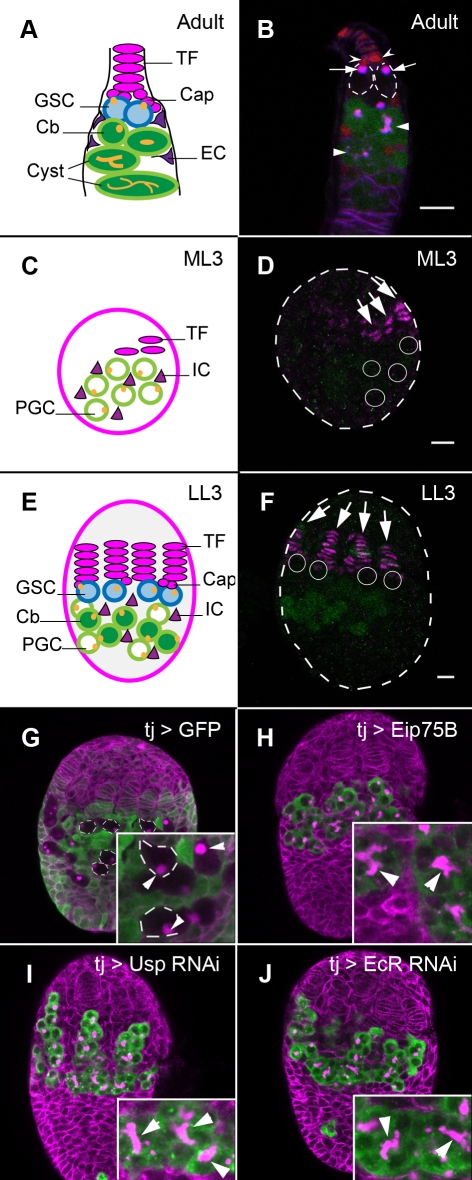
Ecdysone receptors repress precocious PGC differentiation. (A) An illustration of an adult germarium. Niche cells (Terminal Filament, TF, and Cap cells) are at the anterior (magenta). Attached to cap cells are the Germ Line Stem Cells (GSCs, blue). GSC progeny (Cystoblast, Cb; and cysts, green) are posterior to GSCs. GSCs and Cbs carry a round fusome (yellow), while germ line cysts carry a branched fusome. Germ cells are also contacted by somatic Escort Cells (ECs, purple). (B) Adult germarium. TF and cap cells (barbed arrowheads) are marked by *hedgehog-lacZ* (anti β-Galactosidase, red). GSCs (outlined) are attached to cap cells. Fusomes within GSCs are labeled by monoclonal antibody 1B1 (magenta) and are round (arrows). Posterior to GSCs, differentiating germ cells are expressing *bamP*-GFP (green). Fusomes within germ line cysts are extended or branched (arrowheads). (C) An illustration of a larval ovary at mid-3^rd^ instar (ML3). TF cells (magenta) are only beginning to form. Primordial germ cells (PGCs, green) are more posterior. Intermingled Cells (ICs, purple) are associated with PGCs. (D) An ML3 ovary. Terminal filament cells are labeled by anti-Engrailed (En, magenta). Few En expressing cells are present in very short filaments (arrows). Differentiating PGCs are labeled by *bamP*-GFP (anti-GFP, green). PGCs have not yet differentiated, and cannot be recognized by anti-GFP; their location is indicated by white circles. (E) An illustration of a late 3^rd^ (LL3) ovary. Somatic niches (TF, Cap cells, magenta) are marked. GSCs (blue) are established close to TF and Cap cells. Away from niches, PGCs initiate differentiation and some have turned to cystoblasts (dark green). (F) An LL3 ovary. TF stacks and cap cells, marked by anti-En (magenta, arrows), are formed throughout the anterior of the ovary. Many PGCs that are not close to the niches are expressing *bamP*-GFP (anti-GFP, green). PGCs that become GSCs are close to niches. They do not express *bamP*-GFP and their location is indicated by white circles. (G–J) LL3 ovaries, all labeled by 1B1 antibody to outline somatic cells and fusomes within PGCs (magenta). (G) The somatic driver *tj*-Gal4 drives GFP expression (anti-GFP, green) in somatic cells, but not in PGCs (some PGCs are outlined, inset). PGCs in wild-type LL3 ovaries carry round fusomes (inset, arrowheads). (H–J) PGCs are labeled by anti-Vasa (green). Somatic expression of Eip75B (H) or RNAi construct against *usp* (I) or RNAi construct against *EcR* (J) results in formation of multiple cysts harboring branched fusomes (arrowheads, insets). Bars in panel (B), (D), and (F) (for F–J) are 10 µm. Anterior is up.

The Drosophila ovary has been an influential model for understanding the interactions between stem cells and their niches [1],[2]. Each fly ovary contains 16–20 units called ovarioles. At the anterior of each ovariole lies a niche, which is composed of Terminal Filament (TF) and Cap cells (Figure 1A,B). Niche cells produce the ligand Decapentaplegic (Dpp, a BMP2/4 homologue), which acts as a maintenance factor to 2–3 Germ Line Stem Cells (GSCs) that are attached to the cap cells [3],[4]. Dpp signaling within GSCs is required to repress the major differentiation gene *bag of marbles* (*bam*) [5],[6]. When GSCs divide, one daughter cell remains at the niche as a GSC. The second daughter, called a cystoblast, is removed from the niche and initiates the differentiation program by up-regulating *bam*. Germ cell differentiation can be followed by the expression of *bamP*-GFP, a GFP reporter construct that recapitulates Bam expression (Figure 1B) [7]. The cystoblast divides four incomplete divisions to form a 2-, 4-, 8-, and finally a 16-cell cyst. Cyst divisions are coordinated by the fusome, an intracellular organelle that is round in GSCs and extended or branched in germ line cysts (Figure 1A,B) [8],[9].

While much is known about how the GSC unit functions in the adult, how niche precursors and GSC precursors are controlled prior to the formation of the adult GSC unit is less clear. At early larval stages, both gonadal somatic cells (the precursors of niche cells) and Primordial Germ Cells (PGCs, the precursors of GSCs) proliferate. Somatic proliferation at this stage is required to allow correct morphogenesis of 16–20 niches, while PGC proliferation is required to generate sufficient GSC precursors that could occupy the forming niches [10].

At mid third larval instar (ML3), TF differentiation initiates (Figure 1C,D) [11]. TF specification continues throughout the late larval period, and by the late third larval instar (LL3), 16–20 TF stacks have formed (Figure 1E,F) [11]. Cap cells form at the base of TF stacks at LL3. Once TF and Cap cells form, PGCs can attach to them via E-Cadherin, to become the adult GSCs [12]. Excess PGCs that are not attached to Cap cells are not maintained, and differentiate to form the first germ line cysts and egg chambers of the female [13]. While differentiating PGCs express *bam* (Figure 1F), their fusomes are still round (Figure 1G, arrowheads), indicating that they have not divided to form cysts yet.

To maintain PGC proliferation throughout larval development, their premature differentiation is actively repressed. Many of the repressors of PGC differentiation are later required for GSC maintenance; the translational repressors Nanos and Pumilio act in a cell-autonomous manner to repress both PGC and GSC differentiation [14]–[16]. In addition, the somatic cells of the ovary express Dpp. Similar to GSCs, Dpp signaling within PGCs is required for their maintenance [13],[15],[17]. Whether some aspects of PGC maintenance are unique to the precursor cells has not been established. In addition, since both niche and GSC precursors pass through an initial proliferation stage, followed by differentiation, it is unclear whether, or how, those two stages are coordinated between the two populations of cells. Such coordination is required for correct ratios of niches and GSCs, as well as for the correct maintenance of GSCs and their precursors.

In a screen that was designed to find novel regulators of niche and PGC development, we found that target genes of the ecdysone pathway affected PGC maintenance. Ecdysone is a steroid hormone that controls many aspects of larval development, which include temporal control of molting as well as regulating cell fate specification and organ morphogenesis [18],[19]. Ecdysone production in the prothoracic gland is regulated by the brain-derived neuropeptide Prothoracicotropic Hormone (PTTH) [20]. This brain-gland connection is reminiscent of the Hypothalamus-Pituitary link in mammals, which is connected to the gonad in a Hypothalamus-Pituitary-Gonadal (HPG) axis. The HPG axis and hormonal regulation play a major role in the initiation of adult reproduction in mammals. No role for the steroid hormone ecdysone has been suggested in the initiation of oogenesis in flies. However, recent reports demonstrated that ecdysone signaling is required cell autonomously within adult GSCs for their maintenance and non-cell-autonomously within Escort Cells (the somatic cells that contact early germ line cysts, Figure 1A) for correct differentiation of adult GSC daughter cells [21],[22].

We demonstrate that in the fly, a brain-gland-gonad axis exists, and that ecdysone receptors regulate GSC and niche formation. In the first, proliferative, stage of gonadogenesis, ecdysone receptors are required to repress precocious PGC and niche precursor cell differentiation. Later, ecdysone signaling is required for niche differentiation. Finally somatic ecdysone signaling is required to initiate fly oogenesis in a non-autonomous manner. Combined, ecdysone receptors orchestrate the entire sequence of the formation of the GSC unit in the ovary. Other stem cell units might similarly be organized during development.

## Results

### Repression of Precocious Ovarian Development by Ecdysone Receptors

To uncover molecular events that underlie niche formation, PGC maintenance, or their coordination, we performed an over-expression screen in larval ovaries (Supporting Information). The driver line *traffic jam*-Gal4 (*tj*-Gal4), which is expressed in the somatic cells of the ovary, but not in PGCs (Figure 1G), was used to generate non-autonomous effects in PGCs. Such effects require large populations of affected somatic cells and might have been undetected by clonal analysis screens.

Over-expression of two nuclear hormone receptors, Eip75B (Figure 1H) and to a lesser extent Ftz-f1 (unpublished data), in the somatic cells of the ovary resulted in precocious PGC differentiation. In contrast to wild-type ovaries, which contain spherical fusomes (Figure 1G, arrowheads), LL3 ovaries over-expressing Eip75B contained branched fusomes, indicating that PGCs differentiated precociously into germ line cysts (Figure 1H, arrowheads). Eip75B and Ftz-f1 are target genes in the ecdysone response cascade, which times various events throughout embryonic, larval, and pupal life [18],[19]. This cascade initiates when the hormone ecdysone binds to two nuclear receptors: Ecdysone Receptor (EcR) and Ultraspiracle (Usp). Following activation of the EcR/Usp heterodimer, a gene expression program is initiated. Many of the central target genes of this cascade (including *ftz-F1*, *Eip75B* and *broad*) encode transcription factors or nuclear receptors and are common to many tissues. The tissue-specific targets of this signaling pathway are not well characterized.

To test whether precocious PGC differentiation resulted from a change in ecdysone signaling, RNAi constructs against EcR or Usp were expressed using *tj*-Gal4. The ovary-specific expression (henceforth termed “somatic expression”) did not change the timing of the various molting stages, pupation, and hatching. However, extensive differentiation of PGCs was observed in gonads of *EcR* or *usp* RNAi animals (Figure 1I,J, arrowheads). While only 2% of control *tj*>lacZ ovaries contained branched fusomes (*N* = 37), 100% of either *tj*>*EcR* or *usp* RNAi ovaries harbored germ line cysts with branched fusomes (*N* = 77 and *N* = 17, respectively). Somatic expression of different RNAi lines against *EcR* and *usp* all resulted in PGC differentiation (Experimental Procedures).

Recently, ecdysone signaling was shown to maintain adult GSCs in a cell-autonomous manner. To test whether EcR and Usp or their target genes might repress PGC differentiation cell-autonomously, we removed ecdysone signaling components specifically from PGCs. No precocious PGC differentiation was observed when RNAi constructs against *EcR* and *usp*, or a dominant-negative isoform of EcRA (EcRA.W650A, [23]), were expressed using the germ-line-specific driver *nos*-Gal4. Nor was PGC differentiation observed in PGCs mutant for *usp*, *Eip75B*, *Eip74EF*, or *ftz*-*f1* (Figure S1). Broad-mutant ovaries also lacked germ line cysts (see below). Thus, during larval stages, ecdysone receptors in the somatic cells of the ovary are required non-autonomously to repress precocious PGC differentiation.

**Figure 2:**
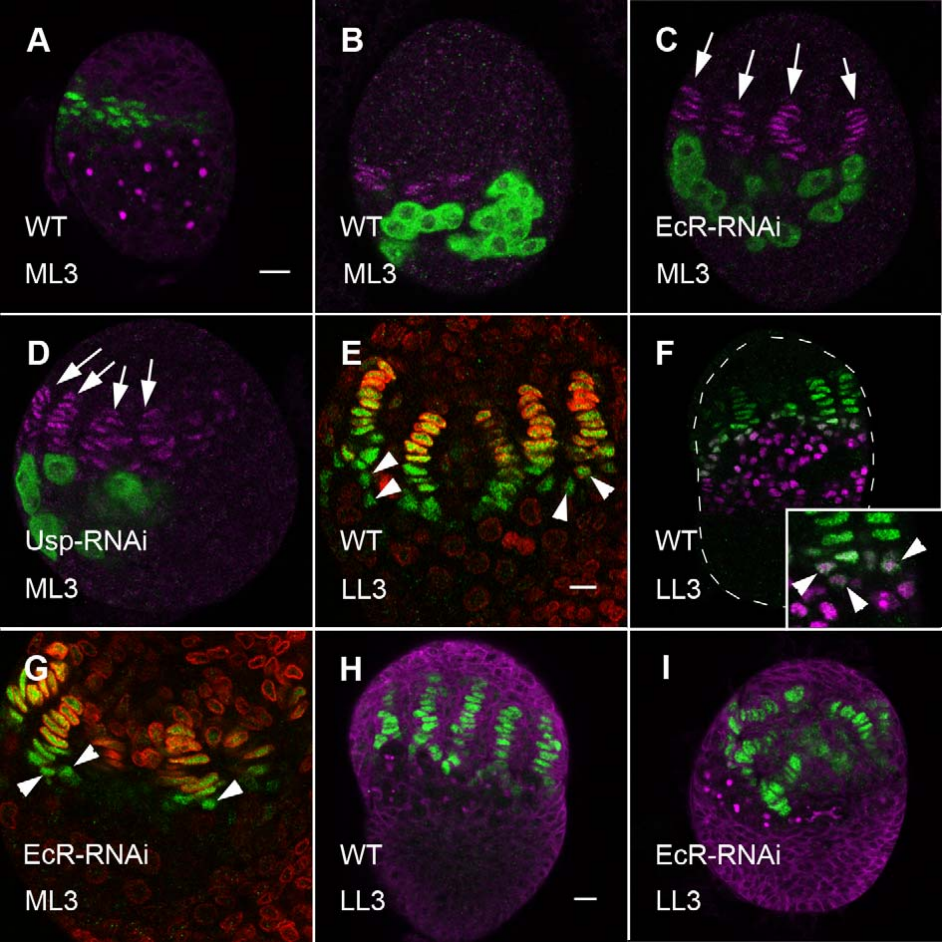
Ecdysone receptors repress precocious niche formation. (A, B) Terminal filaments of wild-type ML3 ovaries are labeled either by *hh*-lacZ (A, green) or anti-En (B, magenta). Few TF cells which are unorganized, or organized into short filaments, can be seen. Germ cells are marked by round fusomes (A, 1B1 antibody, magenta) or by anti-Vasa (B, green). (C, D) In *EcR*-RNAi (C) or *usp*-RNAi (D) ML3 ovaries, more TF cells and more organized filaments can be seen (anti-En, magenta, arrows). Germ cells are labeled by anti-Vasa (green). (E–I) *hh*-lacZ (green) marks all TF and cap cells. (E) In WT LL3 ovaries TF cells are distinguished by *hh*-lacZ staining and oval-shaped nuclei. LaminC (red) is only apparent in older TF cells, at the anterior of each TF stack. Cap cells (arrowheads) are at the posterior base of TF, have rounder nuclei, and do not yet stain with anti-laminC. (F) Anti-Tj (magenta) labels ICs. Cap cells that form at the base of TFs are co-stained with *hh*-LacZ and anti-Tj (inset, arrowheads). (G) *EcR*-RNAi ML3 ovaries. Unlike wild-type, Cap cells appear already at ML3 (arrowheads). (H, I) Somatic cells and fusomes are labeled by 1B1 (magenta). (H) TFs are regularly spaced in the anterior half of the wild-type ovary. (I) In *EcR*-RNAi LL3 ovaries, TFs are mis-positioned, with fewer cells between stacks. Bars in (A), (for A–D), in E (for E, G), and in H (for F, H, I) are 10 µm.

In addition to precocious PGC differentiation, precocious niche differentiation also occurred in *EcR* and *usp* RNAi ovaries. In wild-type ML3 ovaries, only few cells express the TF markers *hedgehog*-LacZ (*hh*-lacZ) and Engrailed (En). These cells are still unorganized, and very few short filaments can be detected at this stage (Figure 2A,B, Table 1). In contrast, removal of *EcR* or *usp* from the somatic cells of the ovary by RNAi resulted in more TF cells, which were already organized into filaments by ML3 (Figure 2C,D, arrows, Table 1).

**Table 1:** Effects of ecdysone signaling components on TF formation.

	Average TF Number	SD	*N*	*t* Test
*UAS*-LacZ ML3	1.8	2.15	41	
*usp*-RNAi ML3	11.29	3.72	28	1.26E-20
*EcR*-RNAi ML3	9.35	4.93	27	1.67E-13
Br-Z1 ML3	ND	ND	ND	ND
Br-Z2 ML3	6.8	1.6	22	1.17E-13
Br-Z3 ML3	6.8	2.2	22	2.39E-12
Br-Z4 ML3	2.8	1.9	24	0.058
LacZ LL3	17.5	2.8	39	
*Eip75B* LL3	13.8	3.5	13	0.000363
*ftz*-f1 LL3	14.7	2.88	20	0.000857

At ML3, TFs were stained with anti-En, confocal Z-stacks were acquired, and the number of TF stacks in ovaries of each genotype counted. An average of about two short TF stacks could be observed in wild-type ovaries. Precocious activation of the ecdysone signaling cascade either by removal of *EcR* or *usp* by RNAi from the somatic cells of the ovary, or by mis-expression of Br-Z2 and Br-Z3, results in more TF stacks at ML3. Very little change in TF stacks is caused by mis-expression of Br-Z4. ND: In Br-Z1 ovaries we cannot quantify TF stacks, since the anterior of the ovary is mis-organized and individual TF stacks are hard to distinguish. However, more TF cells are observed in Br-Z1 ovaries (Figure 5B). At LL3, TF stacks were observed using 1B1 labeling. On average, 17 or 18 TFs are formed in wild type ovaries. When large mutant clones of *Eip75B^07401^* or *ftz-f1^03649^* are generated using *C587*-Gal4, *UAS*-Flp, less TF stacks form.

To test whether all aspects of niche formation were precocious, we examined Cap cells, which appear at the larval-pupal transition stage at the posterior base of TFs [24]. Cap cells contain nuclei that are rounder than TF nuclei and also stain with *hh*-lacZ (Figure 2E, arrowheads). These cells also stain with anti-Tj antibody, which at LL3 stains the Intermingled Cells (ICs, the cells that directly contact PGCs [10],[25]), indicating that cap cells may originate from anterior ICs (Figure 2F, inset, arrowheads). In *EcR* (Figure 2G, arrowheads) and *usp* RNAi (unpublished data) ovaries, cells with cap cell morphology, which were labeled by *hh*-LacZ, appeared at the base of precocious TFs already at ML3. Thus, the development of the entire stem cell niche is precocious when either EcR or Usp are removed from the somatic cells of the ovary. Despite the precocious formation of cap cells in EcR and Usp-RNAi ovaries, we could not observe extra cap cells during larval stages, as has recently been proposed [22]. However, it is possible that increased ecdysone signaling affects cap cell number during pupal or adult stages (Figure S2, Text S1).

Precocious niche development resulted in disorganization of the anterior part of the ovary. In the wild type, niches are formed as well organized TF stacks, which are regularly spaced throughout the anterior part of the LL3 ovary (Figure 2H). In *EcR* (Figure 2I) or *usp* RNAi ovaries (unpublished data), TF stacks formed, but some stacks were not positioned correctly from anterior to posterior. In addition, less non-TF cells were present between stacks and anterior to them (Figure 2I). Since TF and cap cells are post-mitotic, we suggest that their precocious differentiation at the expense of the proliferating precursors caused the reduction in anterior size and resulted in morphogenesis defects.

Despite their spatial disorder, niches had all their cellular components; we therefore tested whether the precocious niches in *EcR* and *usp* RNAi ovaries were functional. Wild type niches secrete Dpp, which results in phosphorylation of Mothers Against Dpp (pMAD, a SMAD homologue) within germ cells that are attached to them. We used immunofluorescence labeling to compare the level of pMAD in PGCs that were close to forming niches in wild type and in *EcR*-RNAi LL3 ovaries. In accord with the normal, albeit early, sequence of niche development, similar levels of pMad were observed in both cases in anterior PGCs that were close to niches (Table 2). Indeed, in *EcR* and *usp* RNAi ovaries, precocious PGC differentiation occurred only in posterior PGCs located away from the niches (Figure 1I,J).

**Table 2:** Effects of abrogation of ecdysone signaling on niche function.

	pMAD Intensity	SD	#PGCs (#ovaries)	*t* Test
*UAS*-LacZ	65.5	11.4	348 (11)	
*EcR*-RNAi	62.6	6.18	397 (16)	0.49
EcRA.W650A	46.3	3.5	267 (15)	2.7E-5

LL3 ovaries were stained with anti-pMAD antibody and scanned in a confocal microscope. Identical settings were used for all ovaries. Intensity levels of pMAD labeling in PGCs that are close to the forming niches were analyzed using the Image J program. No change in pMAD labeling of *EcR*-RNAi ovaries as compared to WT could be observed, indicating that the early forming niches in *EcR*-RNAi ovaries are fully functional. pMAD levels in EcRA.W650A germ cells were reduced, reflecting the general reduced ovarian size, and reduced amounts of niche cells, which produce Dpp.

Taken together, these data show that removing ecdysone receptors from the somatic cells of the ovary leads to precocious differentiation of both niches and PGCs. Forming niches are functional and protect PGCs that attach to them from differentiation. However, the organization of the anterior of the ovary is defective due to precocious precursor differentiation.

### Ecdysone Receptors Are Early Repressors and Late Activators of *broad* Expression

To understand how ecdysone receptors repress precocious niche formation and PGC differentiation, we examined the expression of ecdysone receptors and of the transcription factor Broad, an important target of the pathway. Antibodies directed against EcR-A weakly stained all somatic nuclei in mid and late third instar. EcR-B1 was detected in all somatic nuclei during third instar. As expected, no EcR staining was observed within PGCs (Figure S3). This finding is in accord with the somatic expression of Usp in larval ovaries [26].

**Figure 3:**
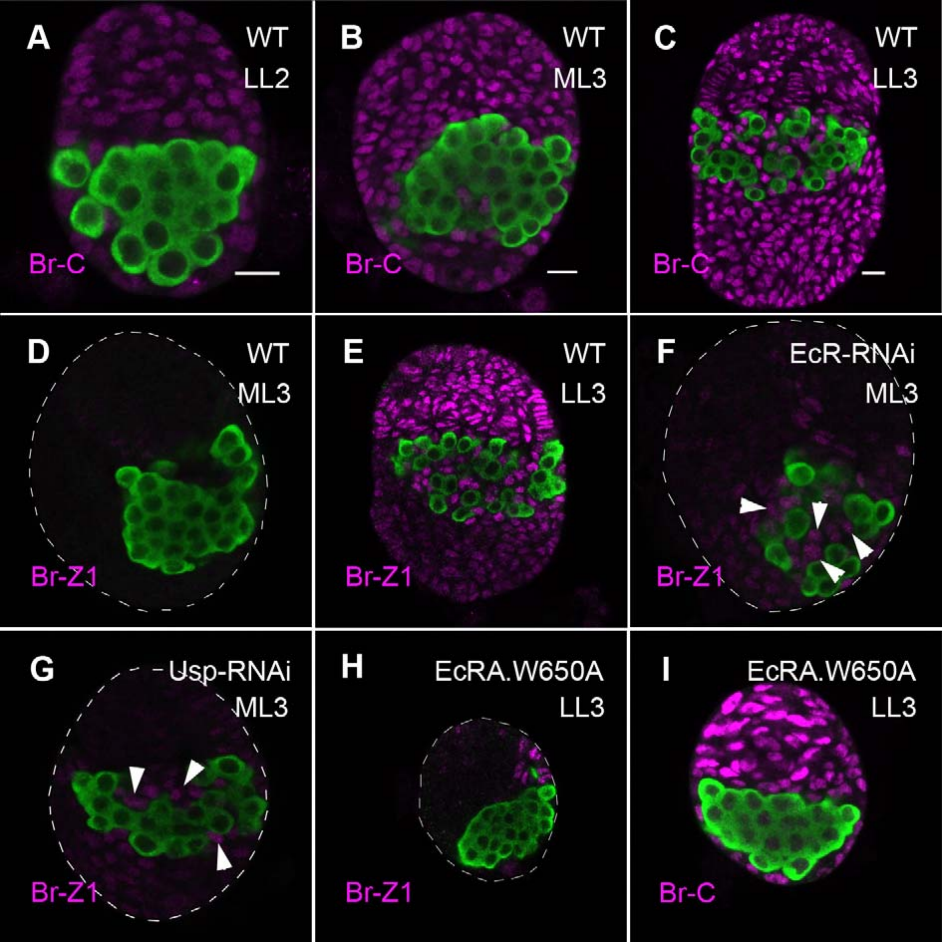
Somatic ecdysone receptors are early repressors and late activators of Broad-Z1. In all panels, germ cells are labeled with anti-Vasa (green). Panels (A–C) were taken with the same confocal settings. Antibodies against the common region of Broad (Broad-C, magenta) stain somatic cell nuclei of LL2 (A), ML3 (B), and LL3 (C) ovaries. Staining levels become stronger with time. Panels (D–G) were taken with the same confocal settings. Broad-Z1 (magenta) is very weakly expressed in wild type ML3 ovaries (D), but is strongly expressed at LL3 (E). In contrast to wild type, Br-Z1 is expressed in ICs (arrowheads) of *EcR*-RNAi (F) and *usp*-RNAi (G) ML3 ovaries. (H) Broad-Z1 (magenta) is not expressed in most somatic cells of EcRA.W650A ovaries. (I) In contrast, anti-Br-C does label somatic cells of EcRA.W650A ovaries. Bars in (A), in (B) (for B, D, F, G), and in (C) (C, E, H, I) are 10 µm.

The *broad* locus encodes four different transcripts: *broad-Z1*, *Z2*, *Z3*, and *Z4* [27]. An antibody directed against the common region of all Broad isoforms exclusively stained somatic cell nuclei. Staining levels increased as ovaries matured (Figure 3A,B,C). One reason for this increase might be the difference observed in the expression of Broad-Z1. Staining with anti-Br-Z1 revealed that this isoform was not expressed until ML3. At ML3 very faint Br-Z1 staining could be observed (Figure 3D), and by LL3 it was strongly expressed in all somatic nuclei (Figure 3E). In contrast, Br-Z1 expression was clearly detected already at ML3 in *EcR* or *usp* RNAi ovaries (Figure 3F,G), suggesting that ecdysone receptors repress early expression of Br-Z1. Significantly, precocious expression was particularly noted in the ICs (Figure 3F,G, arrowheads), which contact PGCs [10],[25].

Inhibition of Br-Z1 expression by EcR was previously observed in imaginal discs [28]. It was suggested that, in analogy to several mammalian nuclear hormone receptors, EcR and Usp have a dual role: in the absence of ecdysone or when associated with co-repressors, these receptors function as repressors of ecdysone target genes, while in the presence of ecdysone or specific co-activators they promote or have a permissive role in target gene activation [28]–[30]. To test this hypothesis, we used a dominant negative isoform of EcR-A, which cannot bind ligand, and serves as a constant repressor [23]. Indeed, Br-Z1 was not expressed, or expressed in very few cells, in LL3 ovaries expressing the dominant negative EcRA.W650A (compare Figure 3H to 3E). These results demonstrate that EcR and Usp act as early repressors of Br-Z1 and that ecdysone signaling is later required for Br-Z1 expression. Anti-Br-C staining was still observed in EcRA.W650A ovaries (Figure 3I), suggesting that Broad Complex is affected by, but not entirely dependent on, ecdysone signaling [31].

### Ecdysone Signaling and Broad Are Required for Niche Formation and PGC Differentiation

**Figure 4:**
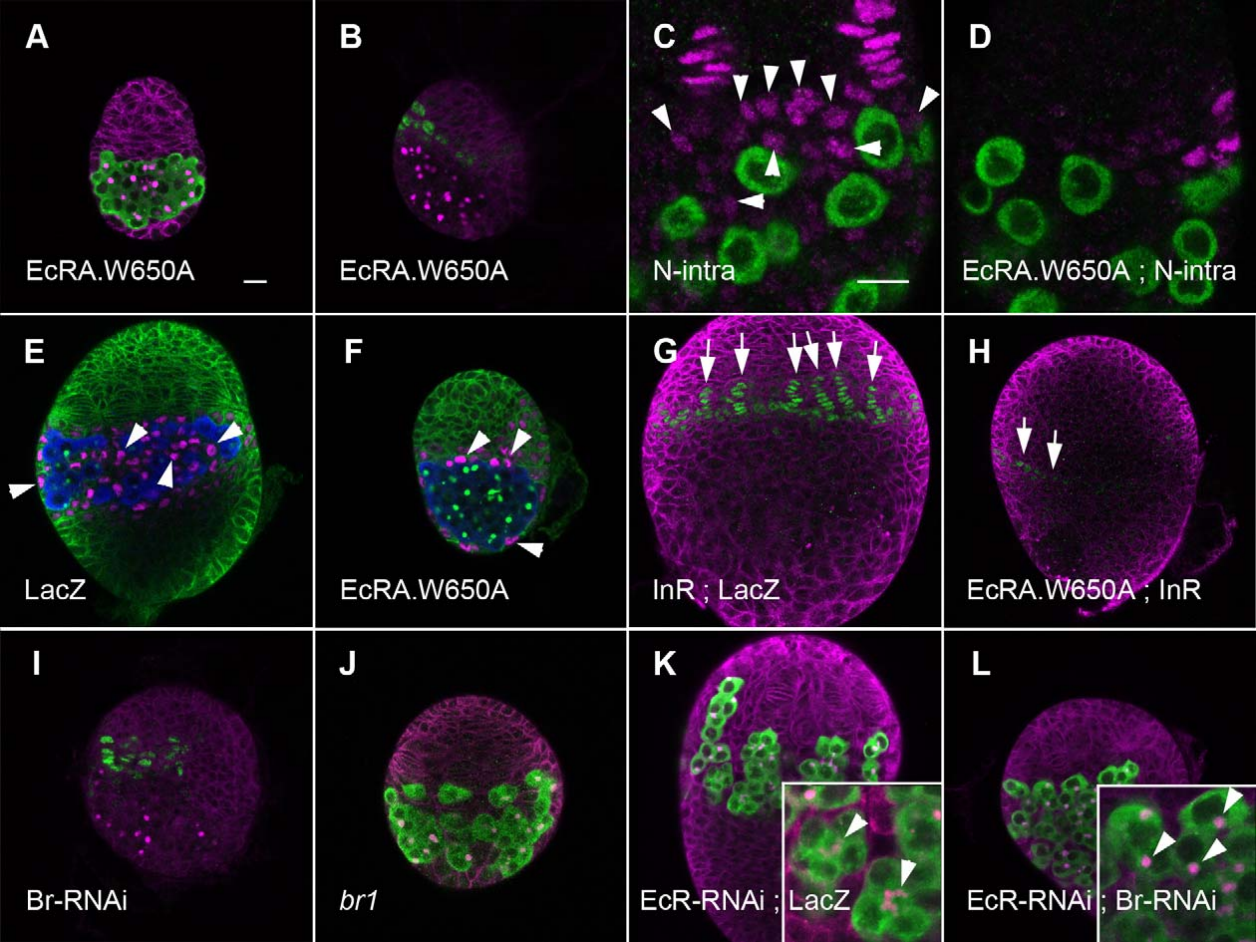
Gonad development requires ecdysone signaling and Broad expression. In all panels except (C, D), 1B1 antibody labels fusomes and outlines somatic cells. (A) Anti-Vasa (green) labels PGCs. EcRA.W650A LL3 ovaries are much smaller than wild type ovaries (compare Figure 4A to Figure 1G). (B) EcRA.W650A ovaries contain very few TF cells (*hh*-LacZ, green), which are not organized into stacks (compare Figure 4B to Figure 2H). (C, D) TF and cap cells are labeled by anti-En (magenta). PGCs are labeled by anti-Vasa (green). (C) Expression of N-intra greatly increases the number of En-labeled cap cells at the base of TFs (arrowheads). (D) Cap cells are not observed in EcRA.W650A ovaries. (E, F) ICs are labeled by anti-Tj (magenta) and are located next to PGCs (anti-Vasa, Blue). 1B1 labeling is green. (E) In wild-type, no Tj labeling is observed at the anterior at LL3. ICs intermingle with PGCs. (F) In EcRA.W650A ovaries, ICs lie outside of the PGC region. As in wild type, no Tj staining is observed at the anterior. (G–I) TFs are labeled by *hh*-lacZ (green). (G) Ovaries over-expressing InR are larger and contain fully formed TF and cap cells (arrows). (H) Over-expression of InR in EcRA.W650A rescues gonadal size. However, very few TF cells are specified (arrows), which are not organized into stacks. (I) Fewer, shorter TFs are present in *br*-RNAi ovaries. (J) *br^1^* LL3 ovaries are small with less developed TFs. (K, L) Precocious cysts and TFs in *EcR*-RNAi ovaries (K, inset, arrowheads) are not observed when ovaries also lack *broad* (I, inset, arrowheads). Bars in (A) (for A, B, E–L) and in C (for C, D) are 10 µm.

Our results suggest that at early third instar, EcR/Usp mediated repression of Br-Z1 expression delays niche and PGC differentiation, while at late third instar, activation of the ecdysone pathway may promote these events by allowing Broad-Z1 expression. To test this hypothesis and determine the role of active ecdysone signaling and Broad expression in the ovary, we expressed the dominant negative form of each of the three EcR isoforms in the somatic cells of the ovary. The dominant negative form EcRA.W650A produced the strongest phenotypes (Figure 4A, Figure S4). EcRA.W650A ovaries were markedly smaller as compared to wild type (100% of the ovaries, *N* = 50, Figure 4A, compare to Figure 1G). Very few TF cells, which were not organized into long stacks, were observed in these ovaries (Figure 4B). It has been previously shown that Notch activation is required for cap cell formation [24],[32]. Indeed, expression of the intracellular portion of Notch in somatic cells markedly increased the number of cap cells forming in wild-type ovaries at LL3 (Figure 4C, arrowheads, *N* = 21). However, cap cells were not induced by Notch activation in EcRA.W650A ovaries (Figure 4D, *N* = 11), suggesting that somatic ecdysone signaling is required to allow Notch-mediated cap cell formation.

The absence of niches in EcRA.W650A ovaries could result from a general developmental arrest, or from a particular problem in niche formation. We therefore tested whether some aspects of gonad morphogenesis did occur properly in EcRA.W650A ovaries. In wild-type ovaries, all somatic cells express the protein Traffic Jam (Tj) until ML3. At this stage the expression of Tj is being limited to ICs [10],[25]. By LL3, only ICs, which intermingle with germ cells, express Tj at high levels (Figure 4E, arrowheads). In EcRA.W650A LL3 ovaries, we found Tj-positive cells in the vicinity of PGCs. These cells failed to intermingle with germ cells (Figure 4F, arrowheads). Significantly, the anterior of the ovary was devoid of Tj protein at this stage, indicating that clearance of Tj from the anterior occurred normally. The fact that not all aspects of ovarian maturation were arrested in EcRA.W650A suggests that ecdysone signaling has a more specific role in niche formation. Indeed, mosaic analysis revealed that less TFs formed in ovaries bearing large mutant clones of Eip75B and Ftz-f1, despite an otherwise normal ovarian development (Table 1).

Their smaller size prompted us to investigate cell proliferation and cell death in EcRA.W650A ovaries. No cell death above wild-type background could be observed (only 0–4 dying cells are observed in WT, *br*-RNAi, or EcRA.W650A ML3 ovaries; *N* = 30, 26, and 42 ovaries, respectively). However, cell proliferation was significantly reduced in *br*-RNAi and EcRA.W650A ML3 ovaries. In LacZ control ovaries, an average of 17.11 cells were labeled with anti-phospho Histone H3, a mitotic marker (SD = 6.99, *N* = 35). Significantly less cells were in mitosis in EcRA.W650A (average of 8.8 cells, SD = 3.47, *N* = 35, *t* test *p* = 2.53E-8) or *br*-RNAi (average 14.2 cells, SD = 4.12, *N* = 50, *t* test *p* = 0.017).

To distinguish between a primary requirement for ecdysone signaling in cell proliferation or in cell differentiation, we forced somatic cells of EcRA.W650A ovaries to proliferate by over-expression of the Insulin receptor (InR). Wild-type ovaries over-expressing InR are larger in size, but their niches are normally patterned (Figure 4G, arrows). In EcRA.W650A ovaries that also expressed InR, ovarian size was similar to that of wild type, indicating that Insulin signaling can overcome the proliferation defect arising from disrupted ecdysone signaling. However, similar to EcRA.W650A, very few TF cells were observed, which were not organized in filaments (compare Figure 4H arrows to 4B and to wild type, Figure 2H). Together with the advanced formation of niches in *EcR* and *usp* RNAi ovaries, these results indicate that ecdysone signaling is required for differentiation of somatic niche cells. In addition, ecdysone signaling may also contribute to somatic cell proliferation [33].

As expected, *br*-RNAi phenotypes were similar to EcRA.W650A in nature but were weaker. *br* -RNAi ovaries were smaller than wild type and had no TFs, or shorter TFs than wild type (100% of the ovaries, *N* = 25, compare Figure 4I to Figure 2H). Similar phenotypes were observed in ovaries from *br^1^* (Figure 4J) or *br^5^* (unpublished data) mutant animals, in which Br-Z2 function is removed (100% of ovaries, *N* = 28 for *br^1^* and *N* = 35 for *br^5^*). Importantly, precocious niche formation and PGC differentiation could not be observed in *EcR* RNAi ovaries that also lacked *broad*. PGCs in such ovaries contained spherical fusomes and TFs were shorter than wild type (Figure 4K,L), suggesting that Broad is an essential component in ecdysone-mediated control of ovarian morphogenesis.

**Figure 5:**
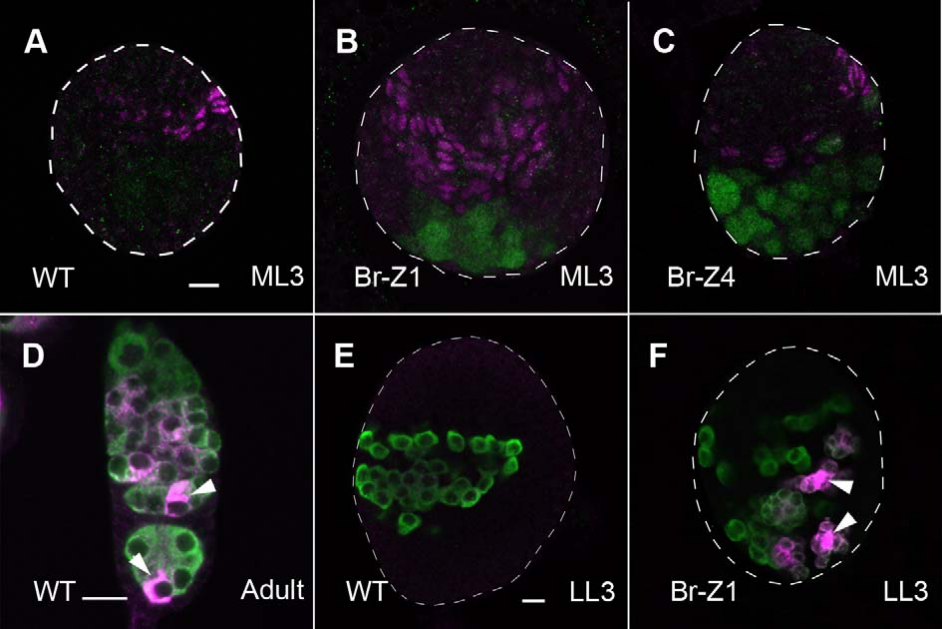
Broad over-expression results in precocious niche and PGC differentiation. (A–C) TF cells are marked by anti-Engrailed (magenta). Differentiating PGCs are marked by *bamP*-GFP (anti-GFP, green). (A) In wild type ML3 ovaries, TF formation initiates, but PGCs are not yet differentiating. No GFP labeling is evident. (B, C) In Br-Z1 (B) and Br-Z4 (C) over-expressing ML3 ovaries, substantial PGC differentiation is observed. More TF cells are specified in Br-Z1, but not in Br-Z4 ovaries. (D–F) PGCs are labeled green (anti-Vasa). (D) In a wild-type adult germarium, 8- and 16-cell cysts are labeled with anti-Orb (magenta). Orb localizes to the selected oocyte (arrowheads). (E) Wild-type PGCs do not express Orb. (F) Cysts in Br-Z1 over-expressing ovaries can express Orb, which is sometimes localized to an oocyte (arrowheads). Bars in (A) (for A–C), in (D), and in (E) (for E, F) are 10 µm.

Our results suggest that removal of *broad* leads to retarded ovarian morphogenesis, while its precocious expression in *EcR* or *usp* RNAi ovaries might lead to advanced morphogenesis and to PGC differentiation. To test this directly we over-expressed each of the Broad isoforms in the somatic cells of the ovary. Niche cells were labeled by anti-Engrailed (En) and PGC differentiation was monitored using the reporter *bamP*-GFP [7]. Over-expression of all Broad isoforms led to precocious *bamP*-GFP expression at ML3 (100% of the ovaries, *N* = 20, 29, 30, and 29 for Br-Z1, Z2, Z3, and Z4, respectively; compare Figure 5A to 5B for Br-Z1, 5C for Br-Z4. Br-Z2, Br-Z3 not shown). Since PGC differentiation was so robust in Broad over-expressing ovaries, we tested the extent to which it could reach. In wild-type adult germaria, Orb is expressed in 8- and 16-cell cysts. When one cell of the 16 is chosen as an oocyte, Orb localizes in this cell (Figure 5D, arrowheads) [34]. As expected, anti-Orb staining of wild type LL3 ovaries revealed no Orb labeling (Figure 5E). However, in Br-Z1 (Figure 5F), Br-Z2, and Br-Z4 (unpublished data) over-expressing ovaries, Orb labeling could clearly be seen. Some cysts already localized Orb into one cell (Figure 5F, arrowheads), indicating that PGC differentiation was advanced and could reach the oocyte determination stage.

TFs also formed precociously following Broad over-expression (compare Figure 5A to Figure 5B, Table 1). Interestingly, while Br-Z1, Z2, and Z3 expression resulted in both precocious TF and PGC differentiation, Br-Z4 over-expression caused only PGC differentiation, but no change in TFs (compare Figure 5A to 5C, Table 1). These results further implicate Broad as a major effector of ovarian morphogenesis, and in particular of niche formation and PGC differentiation.

### Somatic Ecdysone Signaling Affects the Major GSC Maintenance Pathway

**Figure 6:**
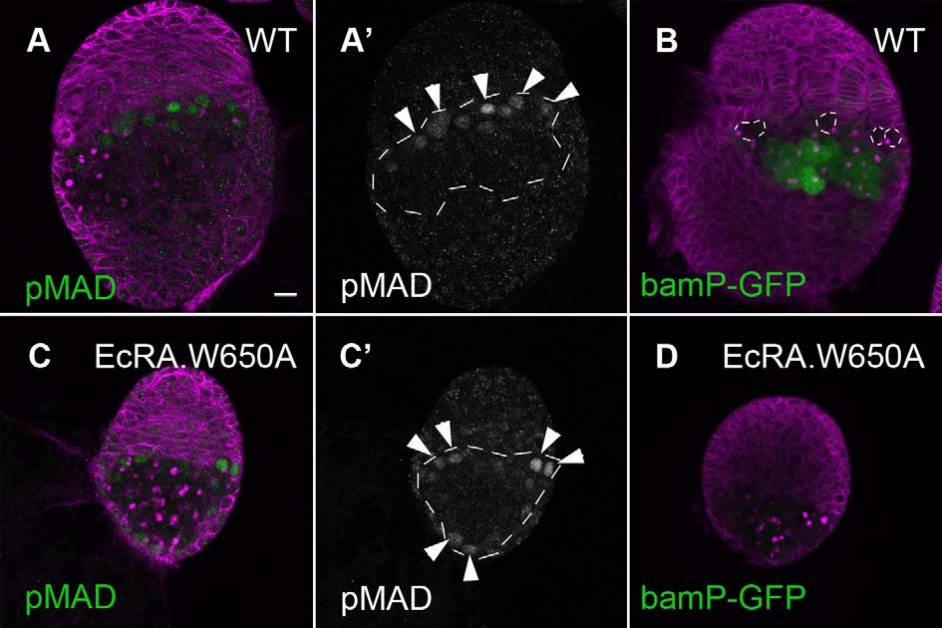
Ecdysone signaling is required for *bam* up-regulation in differentiating PGCs. All panels depict LL3 ovaries. 1B1 (magenta) outlines somatic cells and fusomes. (A) In wild type LL3 ovaries, only PGCs closest to the niche retain pMad labeling (anti-pMad, green). (A′) is a grayscale image of the green channel. PGC region is outlined. pMAD positive PGC nuclei are marked by arrowheads. (B) PGCs that are located away from the niche and do not carry pMAD in their nuclei up-regulate *bam* (*bamP*-GFP, green). PGCs located in niches do not express *bamP*-GFP (outlined). (C) In EcRA.W650A ovaries, some PGCs, which are close to anterior or posterior somatic cells, retain pMAD labeling. Most PGCs do not retain pMad staining. (C′) is a grayscale image of the green channel. PGC region is outlined. pMAD positive nuclei are marked by arrowheads. (D) No corresponding elevation of *bamP*-GFP can be observed in EcRA.W650A ovaries. Bar in (A) for all panels is 10 µm.

To define how somatic ecdysone signaling might induce PGC differentiation, we analyzed its effects on the major germ cell maintenance/differentiation pathway. Similar to GSC maintenance, all PGCs at early larval stages are maintained by Dpp signaling [3],[15],[17], which results in pMad translocation to the nucleus, where it represses *bam* [5],[6]. By LL3, only PGCs that reside at the niche accumulate pMad in their nuclei (Figure 6A,A′ arrowheads). In PGCs that are away from the niche, only background levels of pMad are observed. These PGCs up-regulate *BamP*-GFP (Figure 6B) [7],[13]. Similar to wild-type ovaries, only a fraction of PGCs in EcRA.W650A LL3 ovaries retained pMad, while most PGCs already down-regulated it. We counted an average of 21 (SD = 5, *N* = 15) pMad positive PGCs out of a total of 89 PGCs (SD = 7, *N* = 15). The fraction of pMAD positive PGCs in EcRA.W650A (23.6%) is comparable to the percentage of pMAD positive PGCs in wild type ovaries (45 pMAD positive PGCs, SD = 8, *N* = 11, which are 22.5%–30% out of 150–200 PGCs at LL3).

The spatial distribution of pMad labeling was somewhat different in EcRA.W650A ovaries. pMad labeled cells were located mostly next to the few specified TF cells, but some were also detected at the posterior. pMad-positive PGCs were always in contact with somatic cells (Figure 6C,C′ arrowheads). We assume this difference is due to the fact that ICs, which were shown in the adult to mediate Dpp diffusion [35]–[37], do not intermingle with PGCs in EcRA.W650A ovaries. In addition, pMad levels within PGCs were reduced as compared with wild type PGCs (Table 2), probably reflecting the reduced amounts of niche cells, which produce Dpp [4]. Strikingly, despite the loss of pMad labeling in 76.4% of PGCs, which was comparable to wild type, *bamP*-GFP was not up-regulated in any of these cells (Figure 6D). Thus, although PGCs lose their major maintenance cue, they delay their differentiation in the absence of somatic ecdysone signaling. This result is particularly intriguing since Mad represses *bam* transcription directly [5],[6]. It suggests that PGC maintenance can be uncoupled from PGC differentiation and that other signaling pathways, which are indirectly affected by ecdysone, might integrate on the *bam* promoter.

### Ecdysone Signaling Is Required in Parallel for Niche Formation and PGC Differentiation

The dual effect of ecdysone signaling on both somatic cells and PGCs raises the question of how these two processes are connected. One option is that ecdysone signaling, through broad, is only required for somatic niche maturation, which then triggers PGC differentiation. Alternatively, ecdysone signaling and Broad might be required first for niche formation and later, independently, for PGC differentiation. Over-expression of Broad-Z4 resulted in precocious PGC differentiation, without affecting niche formation, suggesting a separate role for ecdysone in the maturation of these two cell populations (Table 1, Figure 5C).

**Figure 7:**
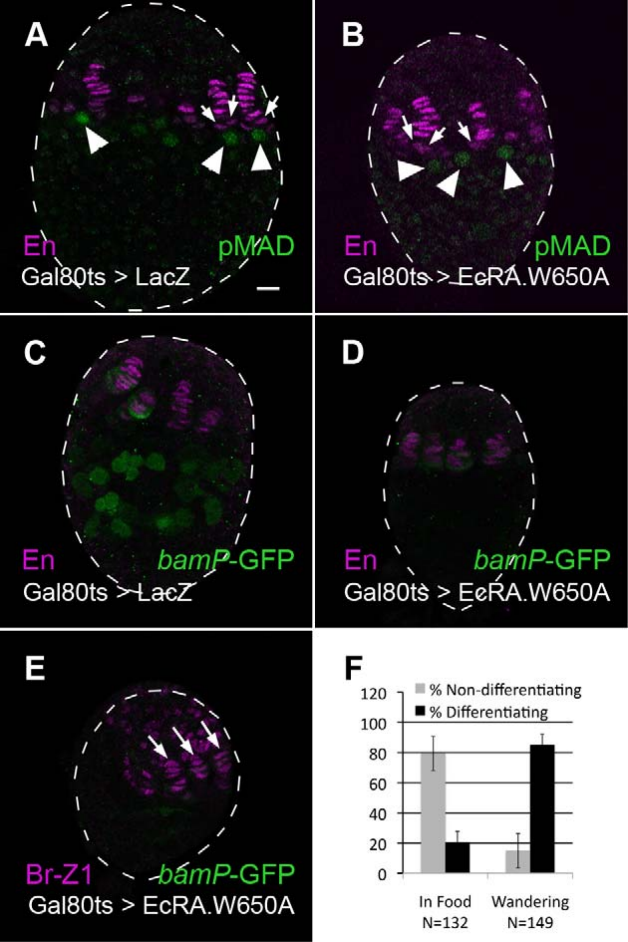
Distinct ecdysone-mediated events are required for niche formation and for PGC differentiation. (A–D) Niche cells are labeled with Anti-En (magenta). (A, B) PGCs containing pMAD (anti-pMAD, green, arrowheads) can be seen in both control and EcRA.W650A ovaries, close to newly formed cap cells (arrows). (C, D) Differentiating PGCs express *bamP*-GFP (anti-GFP, green). (C) In wild-type LL3 ovaries, PGCs that are not associated with niches differentiate and express *bamP*-GFP normally. (D) When ecdysone signaling is blocked by expression of EcRA.W650A after niches form, but prior to PGC differentiation, PGCs fail to differentiate despite normal TF formation. No GFP expression in PGCs is observed. Faint, non-specific, GFP can be observed in a few TF cells in both control and experimental ovaries. (E) Br-Z1 (anti Br-Z1, magenta) is expressed in formed niches of EcRA.W650A temperature-shifted ovaries (arrows), but is not expressed in ICs. Bar in (A) (for A–E) is 10 µm. (F) 0–4 h prior to wandering behavior (in food), most ovaries do not carry differentiating PGCs (light bars), while 0–4 h following the initiation of wandering, most ovaries carry differentiating PGCs (black bars).

To experimentally test whether PGC differentiation depends on an ecdysone-mediated event that is independent of niche formation, we used a temperature-sensitive Gal80 [38] to temporally control the expression of the dominant negative EcRA.W650A. Larvae were raised in a permissive temperature until niche formation had begun, but before PGCs differentiate (Materials and Methods, Figure S5). Following a shift to the restrictive temperature, the state of niche development and PGC differentiation was examined. Under these conditions, TFs and cap cells could be observed in both control and experimental ovaries (Figure 7A,B, arrows). These niches were functional, since PGCs that were attached to them maintained pMAD labeling (Figure 7A,B, arrowheads, *N* = 36 and *N* = 25, respectively). In control ovaries, PGCs that were not located close to niches up-regulated *bamP*-GFP (Figure 7C, *N* = 49). However, PGCs in EcRA.W650A ovaries failed to differentiate and did not up-regulate *bamP*-GFP, despite niche formation (Figure 7D, *N* = 56). Similar results were observed with a temperature-sensitive allelic combination of EcR (EcR^A438T^/EcR^M554fs^, unpublished data, *N* = 25 ovaries). These data suggest that PGC differentiation requires wild-type ecdysone signaling even after niches have formed.

To understand why PGCs failed to differentiate in EcRA.W650A temperature shifted ovaries, we examined Br-Z1 expression. Br-Z1 was expressed in the anterior of these ovaries and in the formed niches (Figure 7E, arrows, *N* = 31). Anterior expression of Br-Z1 in the temperature shift experiments is expected, since in wild-type LL3 ovaries *tj*-Gal4 expression is weak in these regions (Figure 1G). Significantly, no Br-Z1 could be observed in ICs, which are located posterior to the niches, and where *tj*-Gal4 is strongly expressed. These results further implicate Br-Z1 expression within ICs, rather than within niches, as required for PGC differentiation at the end of larval development.

We used the temperature shift approach to further test the temporal requirement of EcR in gonad morphogenesis and found that somatic expression of EcRAW650A only in the adult resulted in normal ovariole morphology (Figure S5). Likewise, a defect in ecdysone signaling during larval development could not be corrected by wild type signaling in the adult ovary. Overall, the temperature shift experiments demonstrated an absolute requirement for somatic ecdysone signaling during larval ovarian development. In particular, these experiments demonstrate that ecdysone is required in parallel for niche and PGC differentiation; even when ovarian morphogenesis is normal, and niches do form, an additional ecdysone-mediated event must occur to allow PGC differentiation.

### A Specific Pulse of Ecdysone Is Required for PGC Differentiation

The temperature shift experiments suggest that PGCs might differentiate in response to a specific ecdysone pulse, occurring after ML3 and prior to pupation. At least one such pulse has been identified in Drosophila [39]. To test this idea more directly, we timed wild-type PGC differentiation by analyzing the expression of *bamP*-GFP and found that PGC differentiation coincides with the initiation of wandering behavior. When insect larvae attain a critical body size, an ecdysone pulse triggers distinct behavioral changes that include cessation of feeding and seeking a location for pupation (wandering behavior) [40]. 0–4 h prior to the initiation of wandering only 21% of the ovaries contained very few differentiating PGCs (Figure 7F). *bamP*-GFP levels in these differentiating PGCs were very low, indicating very early stages of differentiation (Figure S6). In contrast, 0–4 h following the initiation of wandering 85% of larval ovaries contained many differentiating PGCs with strong *bamP*-GFP labeling (Figure 7F, Figure S6). The tight temporal correlation between PGC differentiation and wandering behavior suggests that a specific ecdysone peak is required for PGC differentiation and that hormonal regulation is directly involved in initiating oogenesis in flies.

## Discussion

**Figure 8:**
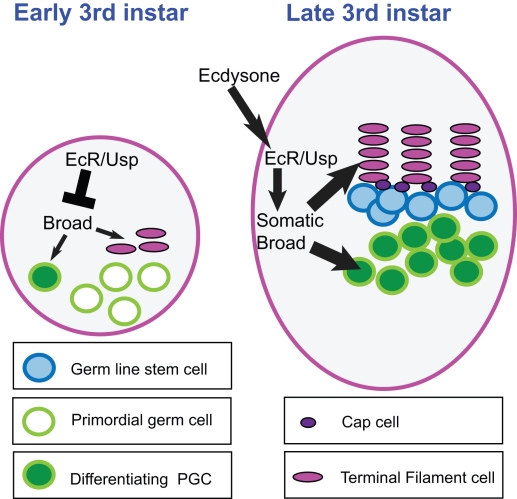
Coordination of niche formation with GSC establishment by ecdysone. At early third instar, ecdysone receptors repress niche formation and PGC differentiation; this allows the gonad time to grow and generate sufficient precursor cells of both cell populations that will eventually form 16–20 stem cell units. Repression of the target gene *broad* is a key component of this repression. At mid-third instar and later, the hormone ecdysone activates Broad-Z1 expression in the somatic cells of the ovary through EcR and Usp. This leads first to formation of niches and, later, concomitant with wandering behavior, to PGC differentiation, by an unknown mechanism. Only PGCs that are located next to niches are protected from differentiation and become the adult GSCs.

When organized niches that contain a defined number of stem cells are established during organ development, the precursors of those niches and stem cells should be coordinated. Such coordination could manifest itself in matching numbers of both populations of cells, and in temporal coordination of proliferation and differentiation. Here we provide evidence that such coordination occurs in the developing fly ovary by ecdysone signaling (Figure 8). To our knowledge, this is a first demonstration that niche precursors and stem cell precursors are coordinated, and that a single signaling pathway is responsible for this coordination. The parsimonious manner of controlling an entire stem cell unit could be a general principle in organogenesis. During organ regeneration, niches and stem cells might also communicate to create well-balanced and morphologically correct stem cell units. Finding the signaling pathways that underlie these processes will prove beneficial for the use of stem cells or their derivatives for organ regeneration.

### The Temporal Axis of Ovary Formation

In the forming Drosophila ovary, the ecdysone signaling pathway coordinates somatic niche formation with GSC establishment, leading to the formation of 16–20 stem cell units. The dual function of early repression and late activation of Broad by EcR/Usp allows this pathway to initially repress both niche and stem cell precursors. Later, ecdysone signaling sequentially initiates TF formation and then PGC differentiation (Figure 8). Within the temporal framework, provided by repeated ecdysone pulses, other signaling pathways may participate in determining the specific rate of precursor cell proliferation and their differentiation. Future work will be needed to determine at what level ecdysone signaling controls these pathways. Our results show that somatic ecdysone signaling elicits a secondary signal that integrates on the major axis of GSC maintenance/differentiation. This signal is required to induce Bam expression in PGCs that are located away from the niche and to initiate their differentiation. Whether ecdysone signaling directly affects the major genes required for niche differentiation remains to be seen.

Ecdysone initiates niche formation at ML3, and PGC differentiation a few hours later. These events do not occur with the earlier peaks of ecdysone, at first and second instar. Gene activation by nuclear hormone receptors is highly context-dependent, and each target gene may require particular co-repressors or co-activators. We hypothesize that the target genes required for the differentiation of niches and PGCs are different, with promoters that require different ligand concentration or different co-activators, which might only be expressed at particular developmental times. Another option (not mutually exclusive) is that the target cells for the two roles of ecdysoene (i.e., niche formation and PGC differentiation) are different; clonal analysis suggests that ecdysone signaling is required within TF precursors for their differentiation, while ICs may control PGC differentiation. Several lines of evidence suggest a parallel role of ecdysone in niche and PGC differentiation. First, over-expression of Broad-Z4 leads to PGC differentiation, without affecting niche formation (Figure 5, Table 1). Second, our temporal shift experiments demonstrated that niche formation in itself is insufficient to induce PGC differentiation (Figure 7). Lastly, in *EcR* and *usp*-RNAi ovaries, Broad-Z1 is over-expressed mainly in ICs, indicating that this cell population, which is in direct contact with PGCs, is a possible source for a signal inducing PGC differentiation (Figure 3). What that substance might be is currently under investigation.

### Changing Roles of Ecdysone Signaling in the Ovary

Activation of the ecdysone signaling pathway in the larva leads to PGC differentiation. In contrast, activation of this pathway in the adult is required to maintain GSCs un-differentiated [21]. Thus, ecdysone signaling serves opposite functions in the adult and in the larva. We have previously demonstrated that many of the mechanisms that maintain GSCs in the adult are already required to maintain PGCs in the larva [15]. Ecdysone signaling is therefore a first regulator that exhibits a reversal of function between a developing stem cell unit and a functional one. The distinct consequence of ecdysone signaling in adult and larval ovaries is reflected in the different manner in which the signal is transmitted. In contrast to the larva, the adult function of ecdysone is cell autonomous and does not seem to strongly rely on Broad function [21]. In addition, somatic ecdysone signaling in the larva is transmitted to PGCs by a signal that integrates downstream of pMad, on the *bam* promoter (Figure 6), while in the adult ecdysone signaling affects GSCs upstream of pMad [21]. In addition to a role within GSCs, ecdysone signaling may be required in Escort cells for correct cyst development [22]. Thus, the different physiological conditions during larval development and in the adult lead to very different effects on a forming versus an adult stem cell unit.

### An Invertebrate Brain-Gland-Gonad Axis

One other difference between adult and larval ecdysone signaling is the source of ecdysone that reaches the ovary. In the adult, ecdysone is produced locally by developed egg chambers and is affecting GSCs in a physiological positive feedback loop [21],[41]. In the larva, developed egg chambers do not exist. The temporal correlation of PGC differentiation with the peak of ecdysone that leads to wandering behavior demonstrates that larval ovaries, similar to other larval organs, respond to ecdysone that is produced by the prothoracic gland, located near the fly's brain.

This suggests a similarity to vertebrate development that was hitherto unappreciated. In verterbrates, a hypothalamic-pituitary-gonadal axis initiates and accompanies adult reproductive responses [42],[43]. This work shows that in fruit flies, a brain-gland-gonad axis also operates. The anatomical analogy, however, does not fully extend to the molecular messengers that convey the signals. The hypothalamus-pituitary connection can be equated with the fly neurons that release PTTH into the prothoracic gland, and elicit ecdysone production [44]. Similar to LH and FSH, which are released from the pituitary gland, ecdysone released from the prothoracic glad affects the gonads and is required for the initial differentiation of PGCs (i.e., for the initiation of oogenesis). Later in adult life, akin to steroid hormones produced by the vertebrate gonad, ecdysone is produced by mature egg chambers [41]. It will be of interest to establish whether the testis in Drosophila males also produces ecdysone.

### Nuclear Hormone Receptors in Organogenesis and Regeneration

Even prior to the initiation of reproduction in mammals, nuclear receptors are involved in gonadogenesis. Nr5a1 is required for the formation of both the ovary and the adrenal gland [45],[46]. Interestingly, Nr5a1 is a mammalian homologue of Ftz-f1, which also has a role in Drosophila gonadogenesis. The physiological role of hormones in niche or stem cell function is not limited to the gonads. Hormones were shown to affect the hematopoietic stem cell niche [47], and the mammalian homologue of EcR promotes neurogenesis in human embryonic stem cell cultures [48]. Steroid hormones are also required for the regeneration of the mammary gland [49],[50]. Similar to our results with ecdysone, the effects of hormones on mammary stem cells are probably indirect, through support cells. Whether the analogy could be extended, and these hormones will prove to affect niche development, remains unanswered. Future work will undoubtedly solve this issue, since understanding how niches and stem cells are coordinated by hormones, or other signals, is crucial for the understanding of regeneration and for applicative approaches in cell-based therapies.

## Materials and Methods

### Fly Stocks

*tj*-Gal4 is a NP insertion line (P{GawB}NP1624) into the *traffic jam* gene, and was obtained from the Drosophila Genetic Resource Centre. *UAS*-Broad-*Z1*, *Z2*, *Z3*, and *Z4* were generously provided by Dr. Lynn Riddiford (HHMI, Janelia Farms Research Campus). *bamP*-GFP is a reporter GFP fused to a fragment of the *bam* promoter. The transgene located on the X chromosome was obtained from Dr. Dennis McKearin. RNAi lines directed against EcR (1765R-4, 1765R-2) or Usp (4380R-1) were obtained from NIG-Fly. RNAi lines against EcR (37058), Usp (16893), and Broad (104648) were obtained from VDRC. RNAi line EcR-IR was from Bloomington. Throughout the main text, RNAi lines 1765R-4, 1765R-2 for EcR, and 4380R-1 for Usp are shown. Somatic expression of *EcR-IR* and line 16893 resulted in fewer and less developed cysts than lines 37058, 1765R-2, 1765R-4, and 4380R-1. FRT19A, *usp^3^* was provided by Dr. Oren Schuldiner (Weizmann Institute). FRT80B, *Eip74EF^DL^* ^-1^ was provided by Dr. Daniela Drummond-Barbosa (Johns Hopkins University). UAS-N^intra^ was provided by Dr. Allison Bardin (Institute Curie). *br^1^*, *br^5^*, *UAS*-EcRA.W650A, *UAS*-EcRB1.W650A, *UAS*-EcRB2.W650A, *Eip75B^07041^*, and *Ftz*-*f1^03649^* were obtained from the Bloomington Stock Center. *UAS*-InR and UAS-lacZ were provided by Dr. Jessica Treisman (NYU School of Medicine).

Somatic clones were generated using the line *c587*-Gal4, *UAS*-flp ;; FRT2A, *ubi*-GFP/TM6. Germ line clones were generated using the line *UAS*-flp; *nos*-Gal4; FRT2A, *ubi*-GFP. *usp* clones were generated using FRT19A, *arm*-lacZ; *hs*-Flp. Clones were induced by heat-shock 48 h AEL, for 30 min at 37°C.

### Larval Staging and Temporal Control of EcR.W650A Expression

To obtain flies in similar developmental stages, care was taken to work with under-crowded cultures. Flies were transferred into a fresh vial to lay eggs for 2 h, and were then removed. Vials were left at 25° for 96 h (mid third instar, ML3) or 120 h (late third instar, LL3). Under these conditions the development of wild type gonads is uniform. The terminology we use is according to Ashburner [51] and is different from the one used by Zhu and Xie [13], who go by King [52].

For time course of PGC differentiation, consecutive layings of 2 h were allowed to mature in a 25°C incubator with 70% humidity and 12 h of dark-light cycles. Under these conditions, flies begin wandering behavior at 112 h AEL.

For temporal control of EcRA.W650A expression: *bamP*-GFP;*tj*-Gal4/UAS-EcRA.W650A;*UAS*-Gal80^ts^, flies were cultured for 6 d at 18°C, then shifted to 29°C for an additional day. Alternatively, a regime of 7 d at 18°C, and a shift to 29°C for an additional day was used (Figure S5). In both cases, larvae were crawling on the bottle walls when dissected.

### Antibody Staining

The following monoclonal antibodies were obtained from the Developmental Studies Hybridoma Bank, developed under the auspices of the NICHD and maintained by the University of Iowa, Department of Biology: Monoclonal 1B1 (developed by Dr. Howard Lipshitz) antibody is directed against an Adducin (1∶20); LC28.26 (contributed by Dr. Paul Fisher) anti-LaminC (1∶20); 6H4 (developed by Dr. Paul Schedl) anti-Orb antibody (1∶20); 25E9.D7 anti-Broad Core (1∶10), Z1.3C11.OA1 anti-Broad Z1 (1∶10) developed by Dr. Greg Guild; 15G1a anti-EcRA (1∶10), AD4.4 anti-EcRB1 (1∶10), AG10.2 anti-EcRC developed by Drs. Carl Thummel and David Hogness; 4D9 anti-Engrailed (1∶20), developed by Dr. Corey Goodman. Rabbit anti-Vasa (1∶5000) was a gift from Dr. Ruth Lehmann (HHMI, New York University). Rabbit anti-pMAD was a gift from Dr. Ed Laufer (Columbia University). Rabbit anti-β Gal (1∶15,000) was from Cappel. Rabbit anti-GFP (1∶1,000) was from Invitrogen. Secondary antibodies were from Jackson Immunoresearch or from Invitrogen.

Unless otherwise specified, all incubations were at room temperature. Ovaries were dissected in Drosophila Ringers Buffer and fixed for 20 min with 5% formaldehyde. Ovaries were then washed once for 10 min with PBS containing 1% Triton-X-100 (1% PBT), and washed again with 1% PBT for an additional hour. Ovaries were blocked with PBS containing 0.3% Triton-X-100 and 1% BSA (0.3% PBTB) for 1 h, and then incubated with first antibody in PBTB overnight at 4°C. Ovaries were washed twice in 0.3% PBTB for 30 min and then blocked with 0.3% PBTB supplemented with 5% Normal Donkey Serum (NDS) for 1 h. Secondary antibody was diluted in 0.3% PBTB supplemented with 5% NDS. Following 2 h incubation with secondary antibody, ovaries were washed three times in 0.3% PBT, 30 min each, and mounted with Vectashield (Vector Laboratories).

Confocal imaging was with Zeiss LSM 710 on a Zeiss Observer Z1.

For statistical analyses, two-tailed student's *t* tests were performed. *p* values are indicated.

## Supporting Information

Figure S1 Manipulation of ecdysone signaling components in PGCs does not induce PGC differentiation. In all panels, 1B1 outlines somatic cells and labels fusomes within germ cells (magenta). (A–D) Germ cells are labeled by anti-Vasa (green). Expression of Eip75B (A), *EcR*-RNAi (B), *usp*-RNAi (C), or the dominant negative EcRA-W650A (D) in germ cells using the germ line driver *nos*-Gal4 does not affect ovary development. TFs form normally, fusomes are spherical or bar-like (indicating dividing PGCs), and no germ line cysts can be observed (insets, arrowheads). (E–G) GFP (green) labels wild-type cells. Germ cells mutant for *Eip75B^07041^* (E), *ftz-f1^03649^* (F), *usp^3^* (G), or *Eip74EF^DL-1^* (H) do not differentiate to form cysts, and harbor spherical or bar-like fusomes (arrowheads). Bar in (A), for all panels is 10 µm.
(TIF)

Figure S2 Different adult expression patterns of Gal4 lines do not correspond to larval expression patterns. In all panels, 1B1 outlines somatic cells and labels fusomes (red). Anti-GFP is green. (A–C) Adult germaria. (A) *ptc*-Gal4 driving UAS-GFP. GFP is expressed in escort cells, but not in cap cells (arrowheads). (B, C) *bab*-Gal4 supports UAS-GFP expression in TF and Cap cells (arrowhead in C). In some ovarioles GFP can also be observed in Escort cells (C). (D, E) LL3 ovaries. In contrast to the different adult expression patterns, *ptc*-Gal4 and *bab*-Gal4 exhibit similar expression patterns in L3 ovaries. GFP is strongly expressed in the anterior of the ovary; weaker expression can be seen in the TF and cap cell region (arrows). ICs are expressing stronger levels of GFP (arrowheads).
(TIF)

Figure S3 Expression of ecdysone receptors in larval ovaries. (A,B) PGCs are labeled by anti-Vasa (green). Anti-EcR-B1 (magenta) stains somatic nuclei at the end of second instar (LL2, panel B). No staining was observed at the end of first instar (LL1), suggesting that EcR-B1 expression is induced during the second instar. (C, E, G, I) *hh*-lacZ (green) stains niche cells to indicate co-labeling with EcRs. (C, D) Low levels of EcR-A (magenta in C, and same image in D, white) are observed in all somatic nuclei at ML3. Earlier expression of EcR-A could not be detected either due to low expression levels or due to low antibody reactivity. Panels (F), (H), and (J) show the indicated EcR labeling in white. EcR-B1 is expressed in all somatic nuclei including forming TFs during ML3 (E, F) and LL3 (I, J). Similar results are obtained with anti-EcR-A (G, H).
(TIF)

Figure S4 Developmental delays upon somatic expression of EcRB1 and EcRB2 dominant negative forms. In all panels, anti-Vasa (green) marks germ cells and 1B1 (magenta) outlines fusomes and somatic cells. (A–C) ML3 ovaries. (A) In wild-type ML3 ovaries, initiation of TF formation can be observed by constriction of cells destined to become TFs (arrow). EcRB1.W650A (B) or EcRB2.W650A (C) ML3 ovaries are smaller in size. In addition, no constriction of TF cells can be observed. In wild type, cells that migrate to the posterior of the ovary are at this stage located medially (A, star). This group of cells is smaller in EcRB1.W650A (B) and hard to find in EcRB2.W650A (C). (D–F) LL3 ovaries. (D) In wild type, TFs are fully formed, and so is the posterior of the ovary (star). (E) In EcRB1.W650A, the posterior group is smaller, while in EcRB2.W650A (F), it is still located medially (star). TFs are smaller and fewer in EcRB2.W650A ovaries. Bars in A (for A–C) and in D (for D–F) are 10 µm.
(TIF)

Figure S5 Temporal requirement for somatic ecdysone signaling. In all panels, germ cells are labeled with anti-Vasa (green). (A–H) 1B1 monoclonal antibody labels somatic cell membranes and fusomes within germ cells (magenta). (A–D) Manipulations were performed using constant expression of *tj*-Gal4 during larval and adult stages (no Gal80^ts^ present). (A) In adult EcR-RNAi ovary niches and cyst development are normal. (B) An entire ovary from EcRA.W650A flies. The somatic expression of this dominant negative construct throughout fly development results in small un-differentiated adult ovaries. No individual ovarioles or normal cyst development could be observed. (C) An entire ovary of a Br-RNAi female. Similar to EcRA.W650A, no individual ovarioles and no proper cyst development could be observed. (D) Br-RNAi is epistatic to EcR-RNAi. Removing both EcR and Br-RNAi results in ovarian phenotypes that are similar to removing Br. (E–J) Temperature shift experiments. Constructs were expressed using a *tj*-Gal4; Gal80^ts^ driver. (E, F) Flies were raised at 18 degrees until adulthood (allowing normal development of niches). Adult flies were shifted to the restrictive temperature for 6 d (KD-knock down). Normal niches and normal cyst development are observed for both control LacZ (E) and EcRA.W650A (F) ovaries, indicating that somatic EcRA does not affect early cyst development in the adult. (G, H) Flies were raised in the restrictive temperature until the end of larval development. Pupae were then transferred to the permissive temperature. While control LacZ ovaries displayed normal oogenesis (G), defective ovaries and lack of oogenesis were observed in EcRA.W650A ovaries. This indicated that the requirement for somatic ecdysone signaling during larval development is absolute and cannot be rescued by normal EcR function in pupal and adult times. (I, J) TF cells are labeled by anti-En (magenta). Larvae were raised at the permissive temperature for 6 (I) or 7 (J) d. TF cells are just beginning to form (I) and first stacks can be seen (J) at these times. Following transfer to the restrictive temperature TF cells still form for several hours, until the effects of Gal80ts wear out (Figure 7).
(TIF)

Figure S6 Temporal sequence of PGC differentiation. In all panels, anti-GFP (green) marks differentiating PGCs and anti-En (magenta) outlines TFs. (A) Representative *bamP*-GFP larval ovary taken from larvae 2–4 h prior to wandering. TF stacks are forming, but PGCs are not yet differentiating. No GFP expression can be observed. (B, C) Ovaries taken from a larvae 0–2 h prior to wandering. Most ovaries still do not harbor differentiating PGCs (B). However, in some ovaries weak GFP expression in very few PGCs can be observed (C, arrowhead). (D, E) Representative *bamP*-GFP larval ovary taken from larvae 0–2 h after wandering behavior is initiated. Many more PGCs are expressing *bamP*-GFP (arrowheads). (F) Ovary taken from a larva 2–4 h following the initiation of wandering behavior.
(TIF)

Text S1 Supplemental experimental procedures.
(DOC)

## Abbreviations

Cb: cystoblast
GSC: germ line stem cell
HPG: hypothalamus pituitary gonadal axis
IC: intermingled cell
LL2: late larval second instar
LL3: late larval third instar
ML3: mid larval third instar
PGC: primordial germ cell
PTTH: prothoracicotropic hormone
TF: terminal filament
