# Supplementary figures and images for: Coordinated Regulation of Niche and Stem Cell Precursors by Hormonal Signaling
Source: PLoS Biol. 2011 Nov 22;9(11):e1001202. doi: 10.1371/journal.pbio.1001202 (PMC3222635; doi:10.1371/journal.pbio.1001202)

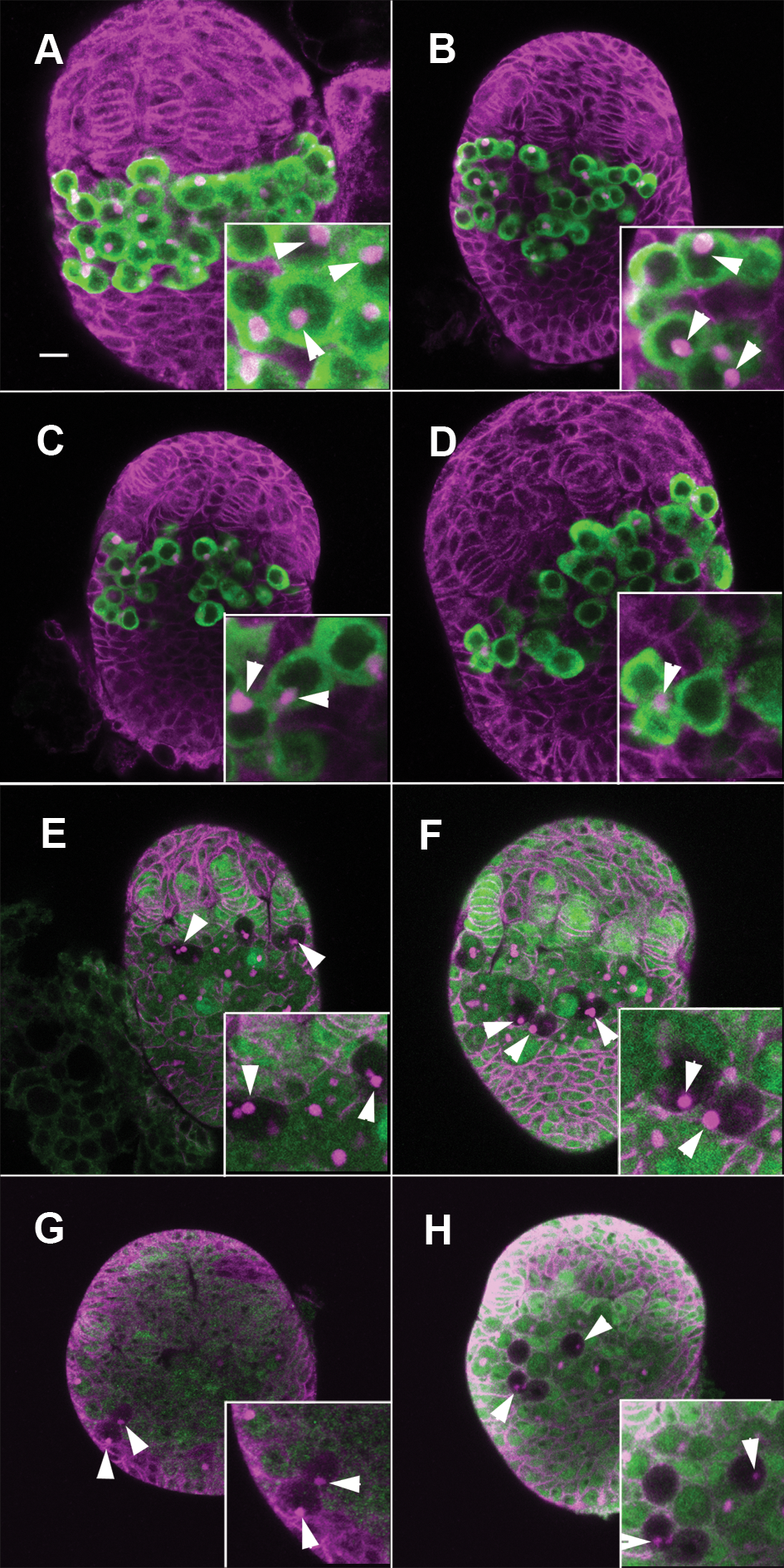

Supplement: Figure S1 — Manipulation of ecdysone signaling components in PGCs does not induce PGC differentiation. In all panels, 1B1 outlines somatic cells and labels fusomes within germ cells (magenta). (A–D) Germ cells are labeled by anti-Vasa (green). Expression of Eip75B (A), EcR-RNAi (B), usp-RNAi (C), or the dominant negative EcRA-W650A (D) in germ cells using the germ line driver nos-Gal4 does not affect ovary development. TFs form normally, fusomes are spherical or bar-like (indicating dividing PGCs), and no germ line cysts can be observed (insets, arrowheads). (E–G) GFP (green) labels wild-type cells. Germ cells mutant for Eip75B07041 (E), ftz-f103649 (F), usp3 (G), or Eip74EFDL-1 (H) do not differentiate to form cysts, and harbor spherical or bar-like fusomes (arrowheads). Bar in (A), for all panels is 10 µm. (TIF) [file pbio.1001202.s001.tif]

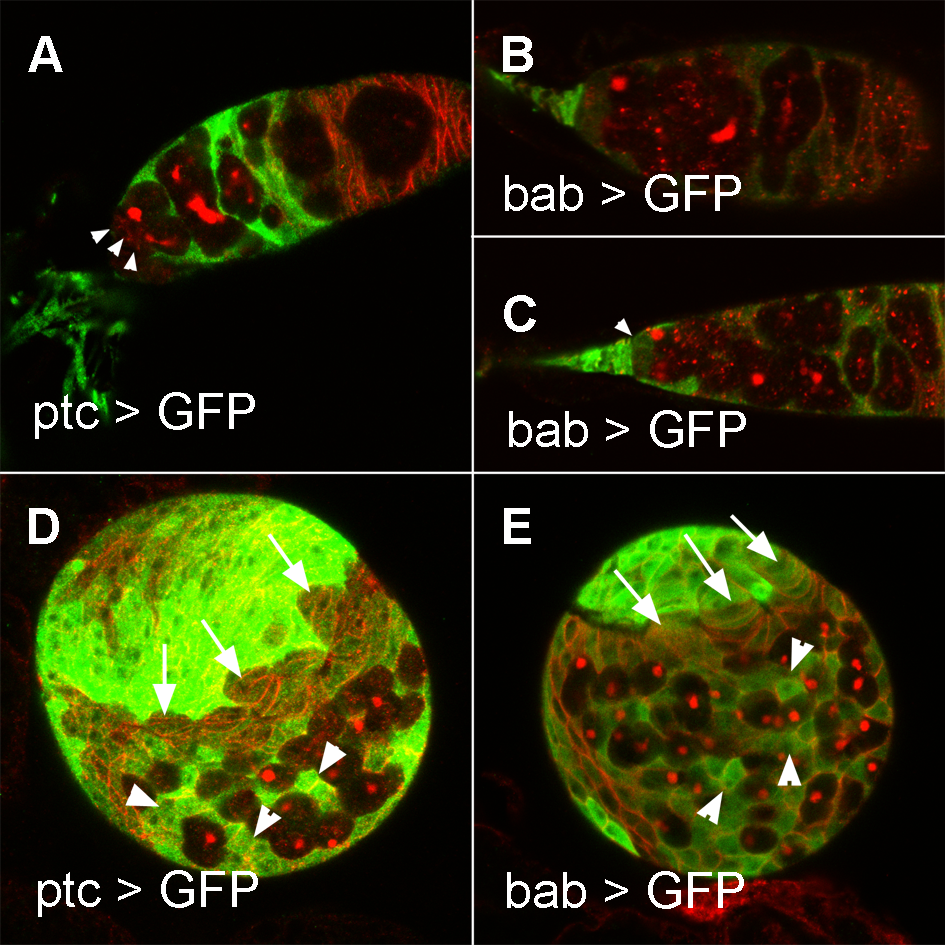

Supplement: Figure S2 — Different adult expression patterns of Gal4 lines do not correspond to larval expression patterns. In all panels, 1B1 outlines somatic cells and labels fusomes (red). Anti-GFP is green. (A–C) Adult germaria. (A) ptc-Gal4 driving UAS-GFP. GFP is expressed in escort cells, but not in cap cells (arrowheads). (B, C) bab-Gal4 supports UAS-GFP expression in TF and Cap cells (arrowhead in C). In some ovarioles GFP can also be observed in Escort cells (C). (D, E) LL3 ovaries. In contrast to the different adult expression patterns, ptc-Gal4 and bab-Gal4 exhibit similar expression patterns in L3 ovaries. GFP is strongly expressed in the anterior of the ovary; weaker expression can be seen in the TF and cap cell region (arrows). ICs are expressing stronger levels of GFP (arrowheads). (TIF) [file pbio.1001202.s002.tif]

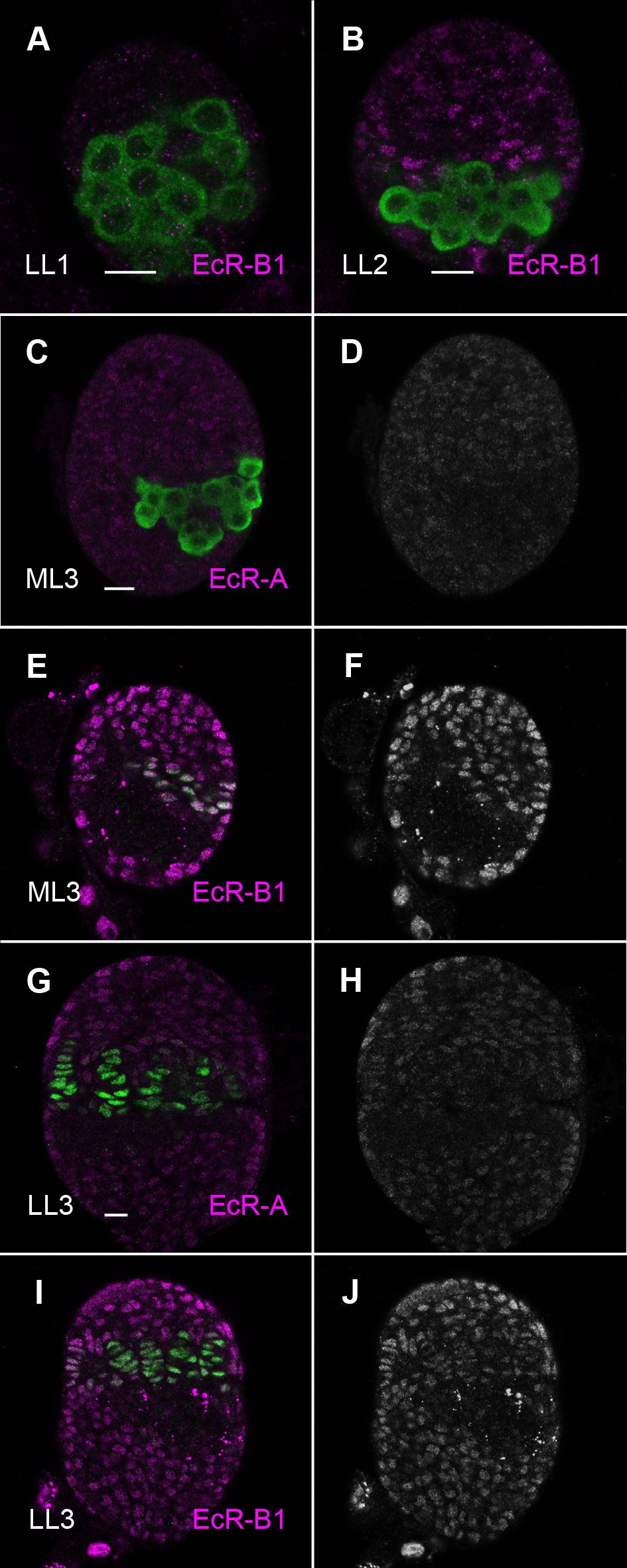

Supplement: Figure S3 — Expression of ecdysone receptors in larval ovaries. (A,B) PGCs are labeled by anti-Vasa (green). Anti-EcR-B1 (magenta) stains somatic nuclei at the end of second instar (LL2, panel B). No staining was observed at the end of first instar (LL1), suggesting that EcR-B1 expression is induced during the second instar. (C, E, G, I) hh-lacZ (green) stains niche cells to indicate co-labeling with EcRs. (C, D) Low levels of EcR-A (magenta in C, and same image in D, white) are observed in all somatic nuclei at ML3. Earlier expression of EcR-A could not be detected either due to low expression levels or due to low antibody reactivity. Panels (F), (H), and (J) show the indicated EcR labeling in white. EcR-B1 is expressed in all somatic nuclei including forming TFs during ML3 (E, F) and LL3 (I, J). Similar results are obtained with anti-EcR-A (G, H). (TIF) [file pbio.1001202.s003.tif]

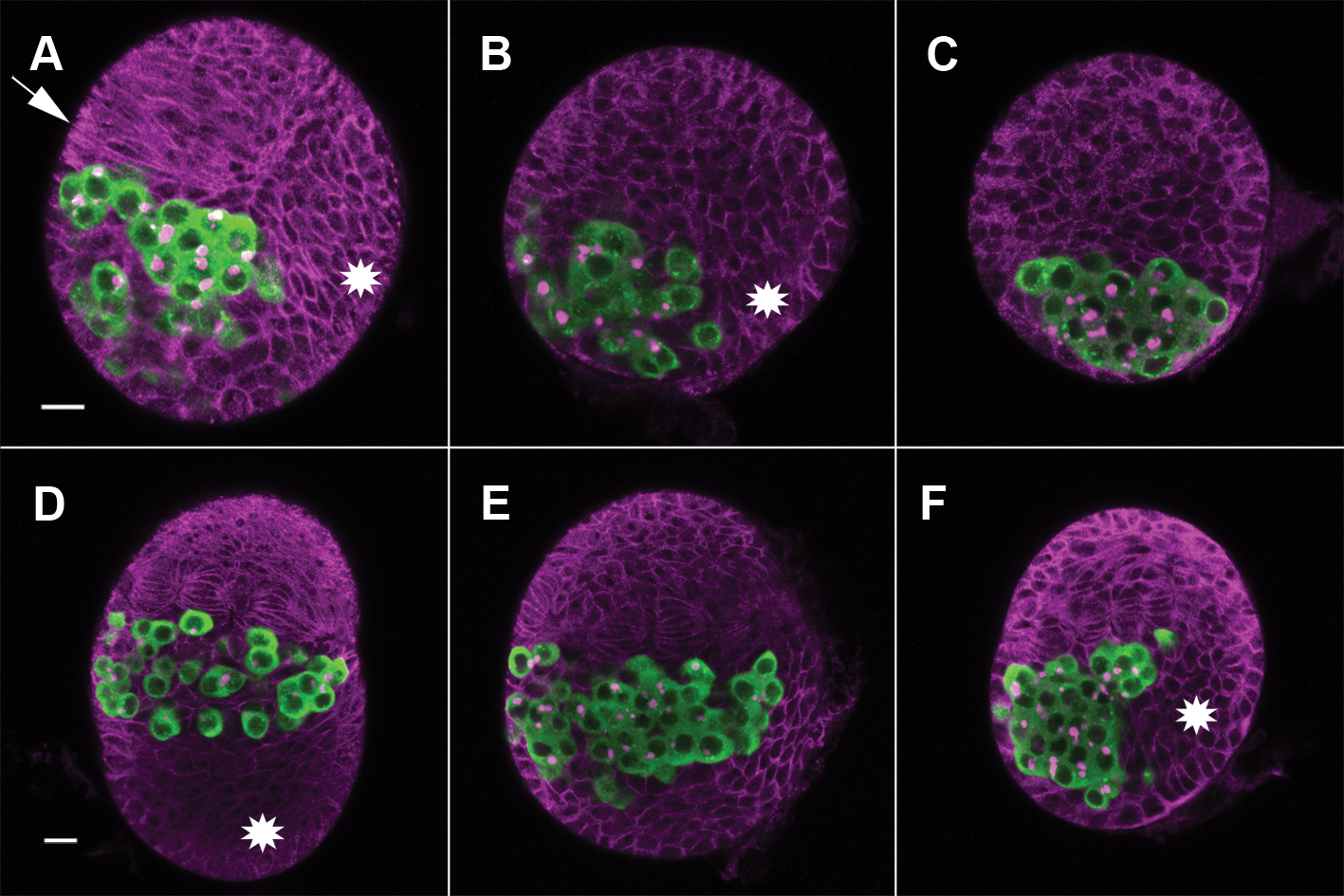

Supplement: Figure S4 — Developmental delays upon somatic expression of EcRB1 and EcRB2 dominant negative forms. In all panels, anti-Vasa (green) marks germ cells and 1B1 (magenta) outlines fusomes and somatic cells. (A–C) ML3 ovaries. (A) In wild-type ML3 ovaries, initiation of TF formation can be observed by constriction of cells destined to become TFs (arrow). EcRB1.W650A (B) or EcRB2.W650A (C) ML3 ovaries are smaller in size. In addition, no constriction of TF cells can be observed. In wild type, cells that migrate to the posterior of the ovary are at this stage located medially (A, star). This group of cells is smaller in EcRB1.W650A (B) and hard to find in EcRB2.W650A (C). (D–F) LL3 ovaries. (D) In wild type, TFs are fully formed, and so is the posterior of the ovary (star). (E) In EcRB1.W650A, the posterior group is smaller, while in EcRB2.W650A (F), it is still located medially (star). TFs are smaller and fewer in EcRB2.W650A ovaries. Bars in A (for A–C) and in D (for D–F) are 10 µm. (TIF) [file pbio.1001202.s004.tif]

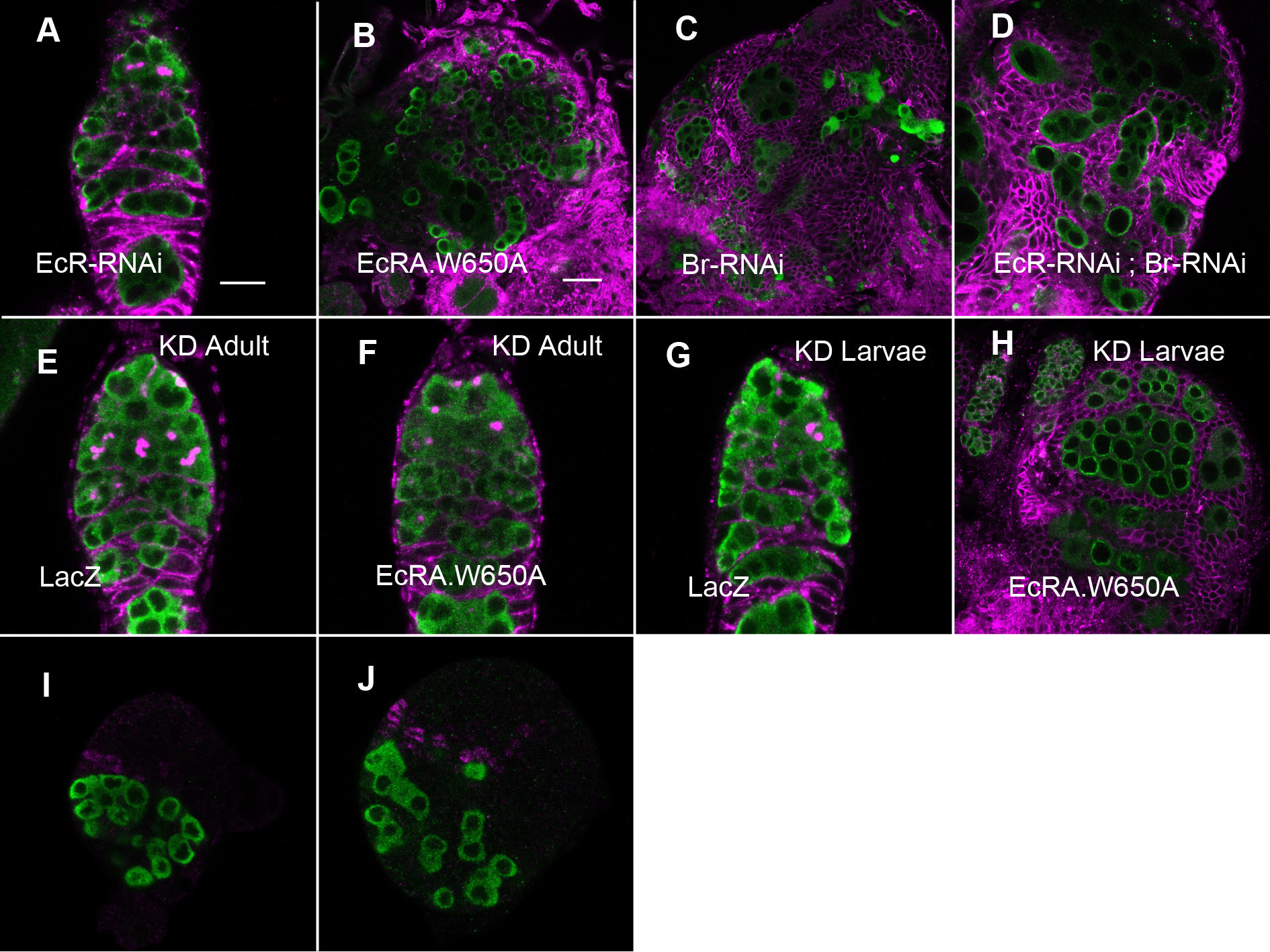

Supplement: Figure S5 — Temporal requirement for somatic ecdysone signaling. In all panels, germ cells are labeled with anti-Vasa (green). (A–H) 1B1 monoclonal antibody labels somatic cell membranes and fusomes within germ cells (magenta). (A–D) Manipulations were performed using constant expression of tj-Gal4 during larval and adult stages (no Gal80ts present). (A) In adult EcR-RNAi ovary niches and cyst development are normal. (B) An entire ovary from EcRA.W650A flies. The somatic expression of this dominant negative construct throughout fly development results in small un-differentiated adult ovaries. No individual ovarioles or normal cyst development could be observed. (C) An entire ovary of a Br-RNAi female. Similar to EcRA.W650A, no individual ovarioles and no proper cyst development could be observed. (D) Br-RNAi is epistatic to EcR-RNAi. Removing both EcR and Br-RNAi results in ovarian phenotypes that are similar to removing Br. (E–J) Temperature shift experiments. Constructs were expressed using a tj-Gal4; Gal80ts driver. (E, F) Flies were raised at 18 degrees until adulthood (allowing normal development of niches). Adult flies were shifted to the restrictive temperature for 6 d (KD-knock down). Normal niches and normal cyst development are observed for both control LacZ (E) and EcRA.W650A (F) ovaries, indicating that somatic EcRA does not affect early cyst development in the adult. (G, H) Flies were raised in the restrictive temperature until the end of larval development. Pupae were then transferred to the permissive temperature. While control LacZ ovaries displayed normal oogenesis (G), defective ovaries and lack of oogenesis were observed in EcRA.W650A ovaries. This indicated that the requirement for somatic ecdysone signaling during larval development is absolute and cannot be rescued by normal EcR function in pupal and adult times. (I, J) TF cells are labeled by anti-En (magenta). Larvae were raised at the permissive temperature for 6 (I) or 7 (J) d. TF cells are just beginn [file pbio.1001202.s005.tif]

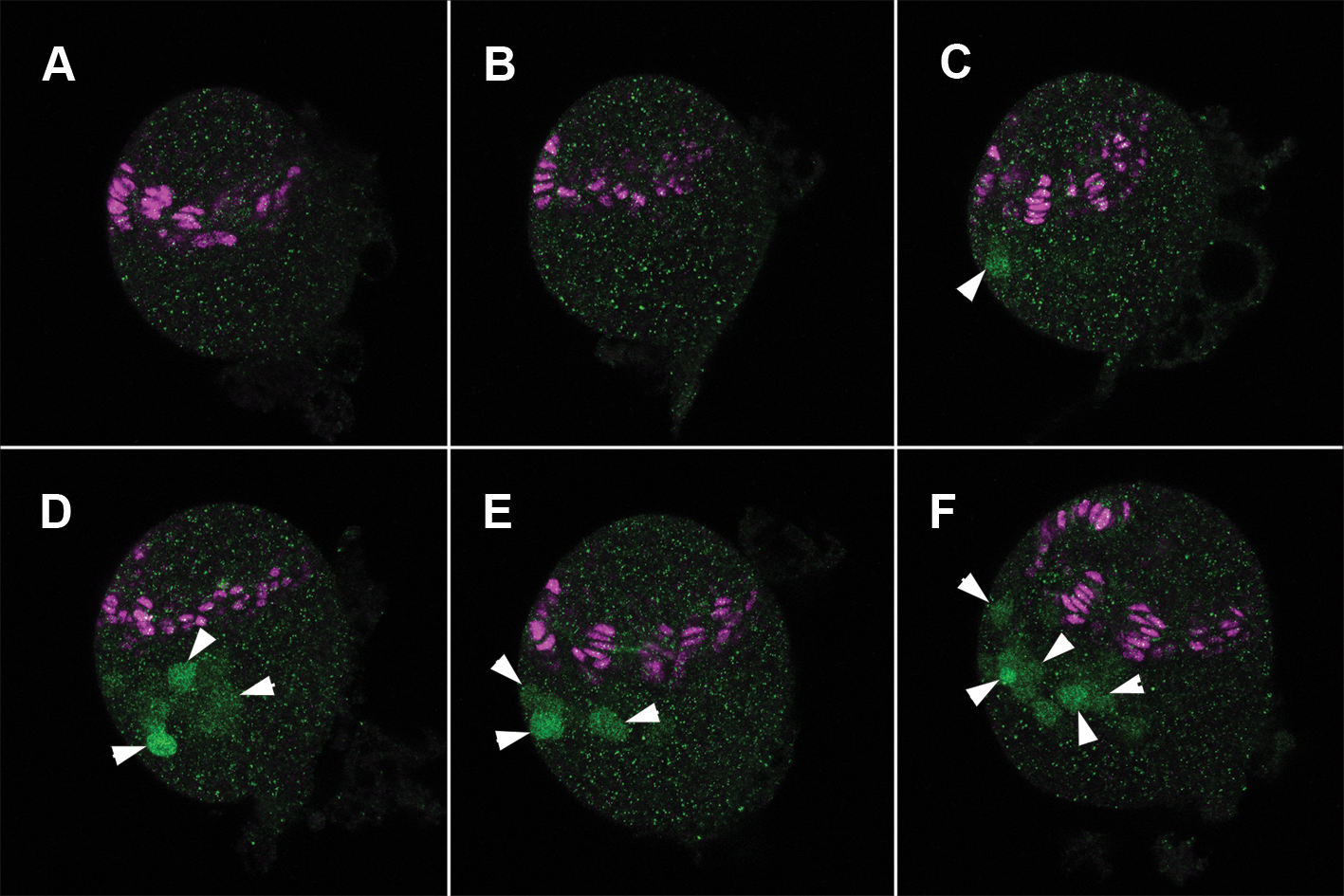

Supplement: Figure S6 — Temporal sequence of PGC differentiation. In all panels, anti-GFP (green) marks differentiating PGCs and anti-En (magenta) outlines TFs. (A) Representative bamP-GFP larval ovary taken from larvae 2–4 h prior to wandering. TF stacks are forming, but PGCs are not yet differentiating. No GFP expression can be observed. (B, C) Ovaries taken from a larvae 0–2 h prior to wandering. Most ovaries still do not harbor differentiating PGCs (B). However, in some ovaries weak GFP expression in very few PGCs can be observed (C, arrowhead). (D, E) Representative bamP-GFP larval ovary taken from larvae 0–2 h after wandering behavior is initiated. Many more PGCs are expressing bamP-GFP (arrowheads). (F) Ovary taken from a larva 2–4 h following the initiation of wandering behavior. (TIF) [file pbio.1001202.s006.tif]
