# Supplementary material for: Coordinated Regulation of Niche and Stem Cell Precursors by Hormonal Signaling
Source: PLoS Biol. 2011 Nov 22;9(11):e1001202. doi: 10.1371/journal.pbio.1001202 (PMC3222635; doi:10.1371/journal.pbio.1001202)
Supplement: Text S1 — Supplemental experimental procedures. (DOC) [file pbio.1001202.s007.doc]

Supplemental Experimental Procedures

Over-expression screen

To uncover additional genes that are involved in larval ovary morphogenesis, PGC proliferation and repression of PGC differentiation, an over-expression screen was performed. The driver line for the screen combined two different Gal4 drivers, *c587*-Gal4, expressed in the somatic cells of the ovary, and *nos*-Gal4, a germ line driver. The driver line was crossed to about 800 lines of the LA collection, generated in Dr. John Merriam’s lab (UCLA). Larvae at late third instar were dissected and stained with 1B1 antibody. This antibody outlines somatic cells and germ cell fusomes. It allows the detection of general morphological defects, changes in PGC number (number of fusomes) and changes in PGC state of differentiation (round vs. branched fusomes).

Lines that displayed a phenotype were subjected to a secondary screen. The secondary screen was performed with two separate Gal4 lines, *tj*-Gal4 or *nos*-Gal4, to ensure that the specific LA line retained the phenotype, and to separate a somatic origin of the phenotype from a germ line origin. A third round of the screen was performed with RNAi lines directed against the over-expressed gene that produced the phenotype.

Of the 800 lines initially screened, 11 genes were previously described that affect GSCs or their niche, and an additional 15 produced strong phenotypes with RNAi and were kept for further study.

Differences in cap cell numbers do not originate during larval development.

A recent paper suggests that increased expression of Ecdysone receptors results in a slight increase in cap cell numbers, and that this change originates during niche formation in the larva [1]. One reason for this suggestion is that when the two drivers *bab*-Gal4 and *ptc*-Gal4 were used to over-express Ecdysone receptors throughout development (i.e. during larval, pupal and adult stages), an increase in cap cells was observed with *bab*-Gal4, but not with *ptc*-Gal4 [1]. These two driver lines are expressed in different patterns in adult germaria, and indeed *bab*-Gal4 is expressed in cap cells while *ptc*-Gal4 is not (Fig. S2 A-C). Since over-expression of Ecdysone receptors was continual from embryogenesis onward, the increase in Cap cells was assumed to originate during niche formation in the larva. However, unlike the adult, during larval development the two driver lines *bab*-Gal4 and *ptc*-Gal4 are expressed similarly (Fig. S2 D, E). This suggests that the increase in cap cells, which was observed at day 7 of adult oogenesis, might have resulted from a change in ovariole structure during pupal or adult oogenesis.

1. Konig A, Yatsenko AS, Weiss M, Shcherbata HR (2011) Ecdysteroids affect Drosophila ovarian stem cell niche formation and early germline differentiation. EMBO J.
